# Isotopic evidence of increased societal diversification in Pre-Columbian Panama

**DOI:** 10.1371/journal.pone.0335678

**Published:** 2025-11-06

**Authors:** Ashley E. Sharpe, Nicole E. Smith-Guzmán, Claudia Patricia Díaz Pérez, Luis Alberto Sánchez Herrera, Diana Carvajal-Contreras, Jason Curtis, Jonathan D. Cybulski, Benoit Desjardins, Ilean Isaza-Aizpurúa, George Kamenov, Yajaira Núñez-Cortés

**Affiliations:** 1 Center for Tropical Paleoecology and Archaeology, Smithsonian Tropical Research Institute, Balboa-Ancón, Panama; 2 Centro de Investigaciones Históricas, Antropológicas y Culturales AIP, Panama, Panama; 3 Sistema Nacional de Investigación, Secretaría Nacional de Ciencia, Tecnología e Innovación, Panama, Panama; 4 Museo Nacional de Costa Rica, San José, Costa Rica; 5 Universidad Externado de Colombia, Bogotá, Colombia; 6 Estación Científica Coiba, Coiba-AIP, Panama City, Panama; 7 Department of Geological Sciences, University of Florida, Gainesville, Florida, United States of America; 8 School of Biological Sciences, Faculty of Science, University of Hong Kong, Hong Kong, China; 9 Department of Anthropology, Université de Montréal, Montreal, Canada; 10 Department of Anthropology, California State University, Sacramento, California, United States of America; University of South Florida, ITALY

## Abstract

The Pre-Columbian history of Panama stands in unique contrast to the state-level societies of Mesoamerica to the north and the Andes to the south. Characterized by a network of powerful chiefdoms at the time of the Spanish arrival in the early 16^th^ century, paleoecological and archaeological evidence indicates that the inhabitants of the isthmus had begun practicing horticulture with early domestic plants by 7000 BCE and adopted ceramic technology around 2500 BCE, both of which were earlier than most of the North and Central American continent. The development of the sociocultural sphere of chiefdoms that arose in this region between 200 BCE – 1500 CE is still not well understood. Focusing on one of the largest sites excavated to date in Panama, Cerro Juan Díaz, this study uses isotopes (carbon, nitrogen, oxygen, and strontium) from bone and tooth enamel of 49 human individuals combined with preexisting isotopic data from nearby sites to understand the diet and mobility patterns of humans in ancient Panama. While the earlier centuries of life at Cerro Juan Díaz are marked by a consistent diet of maize and marine resources among most members of the community, by 700 CE a shift occurs where both diets and movements among individuals become highly variable. By 1150 CE, distinct isotopic differences emerge between sexes and between adults and children, revealing evidence of increasingly diverse social roles and mortuary practices. We interpret these results considering other archaeological and ethnohistoric records from the region to understand social trends that occurred in Panama throughout the 1500 years before Spanish contact.

## Introduction

The rise of increasingly complex social organizations among pre-state and early state societies has been of considerable interest in archaeology for generations [1–4]. It is well known that the distribution of resources and roles within a society evolves over time as a population grows and adapts to the multiple pressures that arise from sustaining an increasing number of individuals on a landscape. In the American tropics, varying forms of political organization arose dependent in part on the local resources available across a physically and biologically complex landscape, and the purposeful acquisition, use, and distribution of these resources [5–13]. While much is known about the state-level societies of Pre-Columbian Mesoamerica and the Andes, the political organization of southern Central America, particularly Panama, is far less understood. Characterized by what has been described as a chiefdom organization at the time of Spanish contact in 1501 CE, settlements in Panama appear to have been led by single individuals, with a loose level of hierarchal organization among the settlements themselves [14–21]. The details of how these chiefdoms operated, including the roles individuals played within a chiefdom and how settlements interacted with one another, are not clearly known since no written records exist from before the Spanish arrived. Indigenous populations were severely affected by disease, war, and forced relocation shortly thereafter [22,23]. Ethnohistoric accounts are few and often focused on matters of interest to the Colonial writers, particularly gold (e.g., correspondence compiled by [24]).

**Fig 1:**
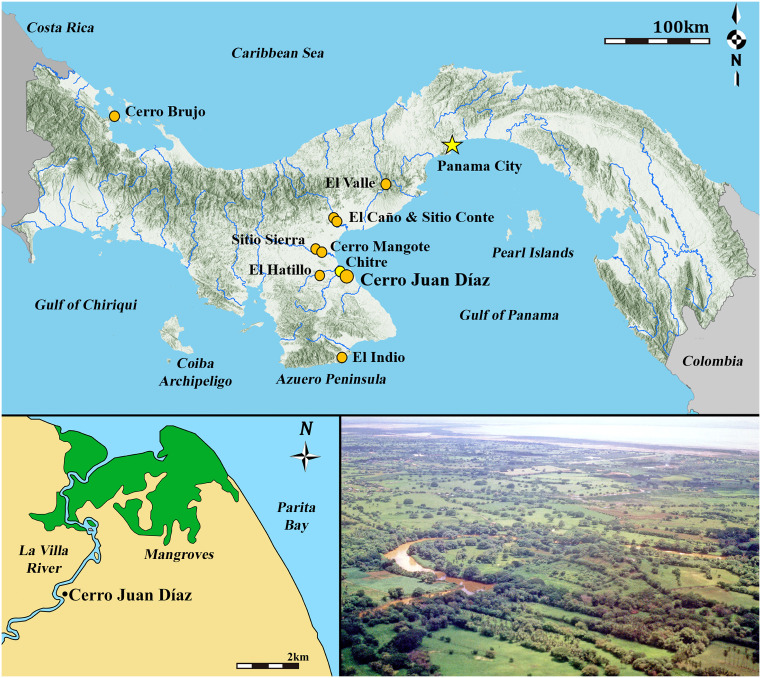
Location of Cerro Juan Díaz, Panama, and other locations mentioned in the text. Archaeological sites in orange, modern cities in yellow. ArcGIS base map by Milton Solano (Esri, ArcGIS Pro version 3.3 and ArcMap 10.8.2) under Creative Commons Attribution License (CC-BY 4.0). River networks were manually digitized from the official 1:50,000 topographic map produced by the Instituto Geográfico Nacional “Tommy Guardia” (IGNTG). Forest cover data correspond to the 2021 national forest cover layer produced by the Ministerio de Ambiente de Panamá (MiAMBIENTE). Elevation shading was derived from JAXA digital elevation model (DEM) to highlight topography. Inset map drawn by Ashley Sharpe in Adobe Illustrator. Aerial photo taken by Richard Cooke in 2000 of the south side of the hill looking toward Parita Bay (Smithsonian Archives, #si_118780, https://researchcomputing.si.edu/exhibitions/).

This study uses a multi-isotopic analysis from humans interred at the large archaeological site of Cerro Juan Díaz (CJD, site designation LS-3; Fig 1; S1 Fig) in central Panama as a means of assessing whether diets and mobility patterns among individuals changed over time as populations in the region increased, driving social transformations. CJD spanned ~150 ha, making it one of the largest sites excavated to date in Panama, and had been continuously occupied from 200 BCE to shortly after the Spanish arrival [25–27]. This period corresponds to the *Gran Coclé Semiotic Tradition*, a time when the various social groups in central Panama shared similar ceramic, metal, stone, and shell-crafting technologies with symbolic traits that were widely recognized throughout the region (S1 Table) [19,28–32]. As this period spans the time of early village settlements to the growth of hierarchical, nucleated chiefdoms, CJD is an ideal site for assessing how human activities changed due to political and economic developments.

Before conducting this study, we hypothesized that if CJD had begun as a low-population village where resources and occupational roles were largely shared by community members, then diets and movements among individuals would be similar. We predicted that we would see more variability in diets and mobility among individuals as chiefdoms arose in the latter half of the first millennium CE, as a result of people taking on more specialized roles within the community, and an uneven distribution of resources as control became more structured concerning their acquisition and use (e.g., Mississippian period Cahokia [33]; Imperial Rome [34]; Zhou Dynasty of China [35]). Our comparison examines differences among individuals of different age-at-death and sex, as well as burial type when possible (e.g., primary, secondary/bundle burial, etc), since all of these may have had a factor in explaining isotopic differences. Results of the study, when compared with other archaeological and ethnohistoric data in the region, show that significant variation in lifestyles and activities among individuals occurred after c. 700 CE.

## Rise of social complexity in Panama

Various lines of material evidence in Panama, primarily ceramic wares, stone and metal technology, and subsistence remains from animal bones, shells, and botanical residues suggest that permanent settlements were uncommon until about two thousand years ago [36]. Paleoecological evidence in the form of plant microfossils indicates that the inhabitants of central Panama had been experimenting with early domestic cultigens (i.e., squash [*Cucurbita* sp.], leren [*Goeppertia allouia*], and arrowroot [*Maranta arundinacea*]) by c. 9000 cal BP [37,38]. Early maize (*Zea mays*) came shortly after, between 7000–8000 BP [37,39,40]. Inhabitants in the region developed ceramic technology around 4500 BP, far earlier than most of the Americas [41,42]. Agriculture and ceramic technology appear to have preceded long-term sedentism by millennia for the isthmian inhabitants.

Throughout most of the Holocene, Panamanian communities appear to have been semi-mobile throughout the year, taking advantage of the seasonal availability of resources from different microhabitats [11,36,43–45]. Historically and even today, members of indigenous communities make seasonal trips to the coast or specific inland locales to fish, hunt, or collect key species or resources [5,46–48]. Certain festivals are also timed throughout the year, such as the *balsería* feasting events among the Ngäbe in western Panama that draw many communities to a single location [49,50]. Based on the predominance of marine sea life depicted in ancient Panama art, as well as the exchange of marine resources from one coast to another (e.g., carved Caribbean manatee bones at sites near the Pacific coast, including El Caño [51] and Sitio Conte [52]), it was likely that most people in Panama knew of the sea, even those who inhabited the interior.

The shift to predominantly permanent settlements around two millennia ago led to a steady growth in village sizes over time, as communities intensified agricultural activities to sustain their populations [14,17–21,36,53]. By around 500 CE, decorated ceramic wares of the Cubitá style began to spread throughout much of Panama, suggesting the expansion of an extensive exchange network [17,25,32,54,55]. In the centuries that followed, gold and other metal ornaments spread with increasing frequency [56]. Clear evidence of social stratification with a paramount ruler is perhaps no more evident than at El Caño and Sitio Conte (700–1000 CE), 50 km north of CJD. These elaborate burial sites are characterized by several large tombs containing hundreds of decorated artifacts alongside interred elites, possibly chiefs or other venerated individuals, accompanied by numerous other individuals whose status is uncertain but may have been of lower rank [51,57–60]. Evidence that high-level chiefs lived near CJD in the La Villa and Parita river valleys comes from both archaeological evidence, such as elaborate burials at El Hatillo [17,21,61] and Spanish historic accounts of the chief domains from the 16^th^ century, particularly that of the chiefs Parita and Escoria who controlled part of the eastern Azuero Peninsula [18–20,25,62,63].

## The Cerro Juan Díaz archaeological site

The focus of the CJD excavations was a natural hill with an elevation of 42 m.a.s.l., located on the southern banks of the La Villa River on the edge of the modern cities of Chitré and La Villa de Los Santos (Fig 1) [25–27]. Surveys in the flood plain around the hill revealed extensive artifactual evidence of occupation on both sides of the river, to an extent of 150 ha [19,20]. Today, the site is located about five kilometers from the mangrove-lined Parita Bay; this distance did not vary significantly in the last two millennia, although the extent of the mangroves may have been greater in the past since shrimp farms and ranches now occupy the region. Excavations at the site were conducted from 1991 to 2001 by local and international archaeologists, focusing on CJD to document what remained of the site after decades of extensive looting and to better understand the history of human activity in the region.

During its occupation, CJD functioned in part as a residential community, possibly due to deliberate selection of its prominent topography and its location along the banks of La Villa River and proximity to the coastline. This positioning provided accessibility for marine resource exploitation and inland trade. Several dozen vertebrate species have been identified at the site, foremost among them white-tailed deer (*Odocoileus virginianus*), Pacific moonfish (*Selene peruviana*), and a variety of catfish (*Ariopsis* sp. *Notarius* sp., *Cathorops* sp.) [43,64]. Faunal remains and isotopic evidence indicate that some CJD inhabitants reared certain birds in captivity, including waterfowl, scarlet macaws (*Ara macao*), and crested guans (*Penelope purpurascens*; S2 Table) [65,66]. There is also substantial evidence for shell harvesting and ornament manufacture, particularly of thorny oysters (*Spondylus* sp.), pearl oysters (*Pinctada mazatlanica*), conch (Strombidae), and ark clams (*Anadara* sp.) [26,67,68]. Surveys conducted on the lower La Villa River valley show that CJD was part of a network of settlements occupied around the same time [19,20]. People were reported living near CJD when the Spanish arrived, and La Villa de Los Santos was founded nearby in the mid-16^th^ century [25,26].

CJD also served as a cemetery for many millennia, and certain areas on and around the hill appear to have been used and reused for burials by multiple generations. The first mortuary horizon was defined by cylindrical pits containing the remains of multiple human individuals dating to the Middle Ceramic Period (200 BCE – 700 CE; S1 Table) that were accompanied by a plethora of some of the most elaborate carved marine shell and metal ornaments at the site, as well as several clusters of perforated carnivore canines and some ceramic vessels [27,69]. The human remains within these burials were distinguished by the following modes: (1) commingled deposits of human remains dating to the La Mula ceramic style (200 BCE – 250 CE) that had been disturbed in antiquity as subsequent burials cut through those originally lain, (2) secondary bundle burials dating to the Tonosí style (250–500 CE), and (3) two primary burials dating to the Tonosí and subsequent Cubitá styles (500–700 CE) [70].

Biocultural indicators on the bones and teeth of individuals buried during the Middle Ceramic Period evidence cultural activities and diseases experienced by these individuals during life. Cultural activity markers included external auditory exostoses (“surfer’s ear”) in individuals who likely engaged in diving activities to recover marine shells for ornamentation and obelionic-type artificial cranial modification that likely served as an embodied marker of sociocultural identity [70–72]. Several individuals also showed signs of health impacts including non-specific inflammation and anemia, as well as probable treponemal disease and developmental anomalies [70,73,74].

Most of these cultural activities and diseases remained prevalent among later burials at the site, dating to the Conte, Macaracas, and Parita ceramic styles of the Late Ceramic Period (700–1400 CE) [69,75]. One glaring exception pertains to the complete absence of external auditory exostoses during this period, which likely relates to the cultural shift from marine shell to gold as the raw material of choice for sumptuary ornament manufacture accompanied by narrowing of maritime trade networks in the Gulf of Panama around 800 CE [30,71,76].

## Methods

### Community engagement and Panama patrimony

Excavations at the Cerro Juan Díaz Project were carried out from 1991 to 2001 with permission of the Panama Ministry of Culture (the National Institute of Culture prior to 2019), under direction of Smithsonian archaeologist Richard Cooke. The project regularly gave excavation tours to the public and school groups during that time, and created exhibits with the nearby museum, the Museo de la Nacionalidad, and the Universidad Santa María La Antigua in La Villa de Los Santos. The human remains recovered from the project have been stored at the Smithsonian Tropical Research Institute’s Naos Archaeological Laboratory with permission from the National Cultural Patrimony department of the Ministry of Culture. Although the ancient inhabitants of CJD have no known direct ancestral ties with any specific indigenous group living in Panama today, the most likely descendants are those people currently living in central Panama and the isthmus, as modern genomic studies have found that most Panamanians share ancestry with Pre-Columbian and modern-day indigenous peoples of Panama [77–80].

Preliminary results from this isotopic analysis and related research on the CJD human remains have been shared to the Panamanian community through the II Congreso de Antropología e Historia de Panamá in Panama City (June 2019), the XIV Congreso Centroamericano de Antropología at the Universidad de Panamá in Panama City (October 2023), a public seminar sponsored by the Secretaría Nacional de Ciencia, Tecnología e Innovación (SENACYT) in Panama City (February 2024), a lecture to geography and history teachers from schools in the interior of Panama (August 2024), a poster session at La Villa de Los Santos to celebrate the Museo de la Nacionalidad’s 50^th^ anniversary (November 2024), in addition to a public talk through the Smithsonian Tropical Research Institute online seminar series (November 2021).

### Bioarchaeological analysis

The organization and analysis of the human remains from CJD has been undertaken by several bioarchaeologists since the 1990s, including Lynette Norr, Claudia Díaz, and Janine Pliska prior to 2016 when Nicole Smith-Guzmán began a project to systematically inventory, analyze, and curate these remains following international standards. Under the latter project, inventory and basic analytic data are recorded and stored in the Smithsonian Institution’s “Osteoware” relational database system (osteoware.si.edu), which follows the North American *Standards for Data Collection from Human Skeletal Remains* [81].

Age and sex estimation of the individuals selected for this study was undertaken by the second author and proceeded as follows. For children (i.e., non-adults between the ages of 0 and 15 years) and older juvenile individuals for whom dental and skeletal development was not yet complete, age was estimated preferentially according to dental development stage [82] and secondarily based on long bone length and epiphyses union [83]. For adults whose dental and skeletal development was complete, age was estimated based on morphological features of the pubic symphysis and auricular surface of the pelvis preferentially [84,85], while cranial suture closure [86,87] was used secondarily, particularly in the absence of observable pelvic features. Sex estimation was only attempted for individuals aged ≥15 years and used pelvic morphology [81,88] preferentially and cranial morphology [86] secondarily.

Due to our requirements regarding sample selection, the majority of the individuals who were tested in this study fall into three chronological groups based on ceramic styles of the Middle and Late Ceramic Periods defined by the Gran Coclé typology (per [89]; S1 Table): Tonosí (250–500 CE), Conte (700–1000 CE), and Parita (1150–1400 CE). Only one individual in the study dated to the La Mula style (200 BCE – 250 CE), and another from the Cubitá style (500–700 CE). Because of this, we combine the La Mula, Tonosí, and Cubitá individuals into a single chronological group, designated the Middle Ceramic Period (200 BCE – 700 CE).

### Stable isotope analysis: Carbon (*δ*^13^C), nitrogen (*δ*^15^N), and oxygen (*δ*^18^O)

A total of 49 individuals were included in this study (Table 1; S3 and S4 Tables). Only burials with secure chronological dates were tested, 18 of which were directly radiocarbon dated. Individuals who had been examined previously by a bioanthropologist (Nicole Smith-Guzmán or Claudia Díaz) were selected for the isotope analysis. We attempted to select individuals when both a tooth and bone sample was available, so as to maintain comparability among isotope values; this was possible in all but five individuals. About 500 mg of bone was obtained for both collagen and apatite analysis, usually from a portion of a femur shaft lacking muscle attachments and other significant anatomical features, and always avoiding metric landmarks. Enamel was selected based on the availability of teeth, as many individuals had missing dentition, or else had unique dental traits that we did not want to alter by sampling. We also attempted to select teeth that had a pair in the dental arcade (e.g., a left maxillary molar was selected if its right maxillary pair was present). In the majority of cases, the enamel from two teeth was selected (first molar and second or third molar, or a premolar), in order to obtain isotope values from different stages during the early life of an individual. In two cases, a canine was selected due to lack of other available teeth to sample, and in three cases a deciduous second molar was selected for the same reason.

**Table 1:** Number of individuals sampled per time period for each isotope.

Chronological Period	Age and Sex	*δ*^13^C_col_, *δ*^13^C_ap_, and *δ*^15^N	*δ*^13^C_en_, *δ*^18^O_en_, ^87^Sr/^86^Sr
Middle Ceramic	Adult Female	7	7 (11 teeth)
	Adult Male	6	7 (17 teeth)
	Child	8	12 (17 teeth)
	Total Middle Ceramic	21	26 (45 teeth)
Conte	Adult Female	5	6 (10 teeth)
	Adult Male	2	3 (6 teeth)
	Child	0	0
	Total Conte	7	9 (16 teeth)
Parita	Adult Female	5	4 (7 teeth)
	Adult Male	4	4 (9 teeth)
	Adult Undetermined	1	1 (1 tooth)
	Child	4	4 (6 teeth)
	Total Parita	14	13 (23 teeth)
	Grand Total	42	48 (84 teeth)

See S3 Table and S4 Table for detailed information about bone and teeth data, respectively. Note that bone collagen and apatite values for Ind. 9 (adult male, Middle Ceramic), Ind. 26 (adult male, Conte), and Ind. 31 (adult female, Conte) were excluded from this list due to poor preservation.

In humans, the permanent first molar crowns develop during the first four years of life [82]. They are roughly comparable to the deciduous second molar, which begins developing in-utero and has completed crown development by the second year of life. The crowns of the permanent canines, premolars, and second molars develop between the second and ninth years, and their isotope values are representative of the individual’s early childhood years. Canines, premolars, and second molars are considered to represent comparable ages in this study. The third molar crown develops between the seventh and sixteenth year, representing the later childhood and adolescence of an individual.

Photographs were taken of each skeletal or dental element prior to testing, and complete measurements were taken of every tooth and bone using standard techniques [81,90]. All analyzed skeletal elements and teeth had already undergone osteological analysis prior to the study, including inventory, assessment of demographic features (i.e., age, sex, and stature), and documentation of pathologies, metric and non-metric traits, and anomalies for each individual. A Dedeco carbide drill was used to sample ~500 mg of bone, with a preference for central cortical shafts of femurs. A ~ 30–50 mg piece of enamel was taken from each tooth in a clear area, usually the buccal or lingual side, using a Dedeco carbide blade. Each piece was cleaned under a Leica S6D stereo microscope with a Brasseler Forza L50K dental drill to make sure no extraneous particles (i.e., soil) or dentin were on the surface, and the piece was divided for strontium and carbon/oxygen isotope analysis. Bulk sampling was performed in this analysis as opposed to serial (intra-tooth) sampling due to sampling restrictions. However, bulk sampling is not intended to compare specific changes in the diet during the growth of an individual tooth [91–93], and intra-tooth sampling is an area for future research in Panama.

Pretreatment of samples took place at the isotope preparation laboratories of the Smithsonian Tropical Research Institute’s Center for Tropical Paleoecology and Archaeology (STRI-CTPA). Bone samples were precleaned using gentle manual scraping with a dental pick, and then washed with distilled-deionized water (DI-H_2_O) in a sonicator bath for approximately 10–15 minutes. Each sample was dried, then ground with a ceramic mortar and pestle, using a different sterilized set for each sample. Bone was sieved through two meshes to separate fractions for collagen (0.25–0.50 mm) and apatite (<0.25 mm) analysis. Sample pretreatment for collagen followed methods described by [94] and slightly amended in [95]. Specifically, approximately ~300 mg of bone sieved for collagen was placed in a sterilized centrifuge tube for the collagen analysis, and 12 ml of 0.2 M hydrochloric acid (HCl) was added to each sample. Samples were gently agitated and left for ~16 hours, then centrifuged and the acid replaced until the bone had demineralized (~2–5 days). Afterward, samples were rinsed to neutral pH with DI-H_2_O, and 12 ml of 0.125 M sodium hydroxide (NaOH) was added. After 16 hours the samples were centrifuged and rinsed to neutral pH again with DI-H_2_O, and then placed in a sterile glass scintillation vial in an oven at 95°C with 10 ml of 1.0 x 10^-3^ HCl for 4–5 hours. About 100 ųl of 1.0 M HCl was then added to each sample and they were returned to the oven for another 4–5 hours in order to assist in the dissolution of soluble collagen [94]. Samples were then returned to the centrifuge tubes, centrifuged, and the solution (not precipitate) was pipetted out to the scintillation vials to be evaporated in the oven at 65°C until each sample had ~ 2 ml. Samples were then capped and frozen, then freeze-dried for two days and weighed for collagen yield (S3 Table) on a Torbal AGN200 analytical balance.

Samples were exported with Panama Ministry of Culture (Ministerio de Cultura de Panamá) permit N°072−21 DNPC to the University of Florida Department of Geological Sciences Light Stable Isotope Laboratory and analyzed for *δ*^13^C and *δ*^15^N on a CarloErba elemental analyzer connected to a Thermo Electron DeltaV Advantage isotope ratio mass spectrometer. *δ*^13^C_co_ was compared against the standard Vienna Pee Dee Belemnite (VPDB), and *δ*^15^N values were compared with atmospheric nitrogen (AIR), primarily using USGS40. Precision for *δ*^13^C_co_ was 0.06‰ and *δ*^15^N was 0.09‰ (n = 6). Detailed information regarding laboratory standards and precision is included in (S5 Table). Approximately 0.6–0.8 mg of collagen was used for isotopic and %C and %N analysis.

Collagen yields, %C, and %N are reported in the S3 Table. Acceptable collagen yields for archaeological, non-modern bone sometimes vary in reported analyses (see [96] for a summary), but generally >0.5–2.0% of the original bone weight is considered acceptable, with lower values indicating degradation that could affect the isotopic results [97]. Values for %C and %N are considered acceptable above 13% and 4%, respectively [98]. The atomic C:N is another common means of assessing whether the collagen in a sample is degraded or contaminated; values between 2.9–3.6 are generally considered acceptable [98,99]. As there are many factors that can influence degradation and contamination of collagen, including preservation conditions, age of sample, and laboratory methods, a consideration of each of these criteria (collagen yield, %C, %N, and C:N) together rather than reliance on a single criterion for quality control is recommended for determining if a sample’s collagen is too degraded for accepting isotope results [96,97].

The bone fraction to be used for apatite analysis was added to separate 15 ml centrifuge tubes, and 12 ml of 2.5% sodium hypochlorite (NaOCl) was added to each (based on [100]). Samples were left for 16 hours with occasional light agitation, and then rinsed to neutral pH. Approximately 12 ml of 0.2 M acetic acid (CH_3_COOH) was added to each sample, and they were allowed to sit for 16 hours before being rinsed to neutral pH. Samples were then frozen and freeze-dried for two days, then sent to the University of Florida Department of Geological Sciences Light Stable Isotope Laboratory under the same permit as above and weighed and analyzed on a Kiel III carbonate prep device connected to a Finnegan MAT 252 isotope ratio mass spectrometer. Precision using the NBS 19 standard was 0.02‰ for *δ*^13^C_ap_ and 0.04‰ for *δ*^18^O_ap_ (n = 16; see S5 Table for information regarding laboratory standards and precision). Bone *δ*^18^O_ap_ is not used in this study for tracking mobility, because it is prone to diagenetic alteration [100].

Enamel samples were ground with individual, sterilized agate mortars and pestles. Samples were divided for either carbonate analysis or strontium and trace element analysis (each ~15–25 mg). Enamel samples tested for carbonate isotopes were pretreated using the same process as the bone apatite fraction, except using microcentrifuge tubes due to the smaller sample size. The same Kiel III carbonate prep device and Finnegan MAT 252 isotope ratio mass spectrometer were used as had been done for the bone apatite. Precision for the enamel apatite samples using the NBS 19 standard was 0.02‰ for *δ*^13^C_en_ and 0.04‰ for *δ*^18^O_en_ (n = 24; S5 Table).

### Dental enamel strontium isotope analysis (^87^Sr/^86^Sr) and trace element concentrations

Enamel samples analyzed for strontium and trace elements were prepared at the Class 100 Clean Laboratory of the University of Florida Department of Geological Sciences. Specimens were dissolved in 2 ml of 8 N nitric acid (HNO_3_) in individual, precleaned Teflon vials. Samples were evaporated on a hot plate at 100ºC, and then a solution of 5% HNO_3_ spiked with 8 ppb Re and Rh was added to each sample according to sample weight to achieve a dilution of 2000x. The dissolved samples were capped and heated for ~4 hours. A small quantity (~100 ųl, depending on dilution) of each sample was extracted in order to conduct the trace element concentration analysis, using a Thermo Scientific Element2 HR-ICP-MS, following methods in [101]. The remaining solution to be tested for strontium was put on a hot plate at 100ºC and allowed to evaporate. Strontium was separated using ion chromatography, using strontium-selective crown ether resin (Sr-spec; Eichrom Technologies) with multiple washes of 3.5 N HNO_3_ [102]. Strontium isotopes were measured on a Nu Plasma multiple-collector inductively coupled plasma mass spectrometer (MC-ICP-MS). All isotopes were measured on Faraday detectors in static mode. Samples were introduced to the MC-ICP-MS in 2% HNO_3_ (Optima). On-peak zero was measured on pure 2% HNO_3_ before each sample introduction in order to correct for isobaric interferences caused by impurities of Kr in the Ar carrier gas. ^87^Sr/^86^Sr was corrected for mass-bias using exponential law and ^86^Sr/^88^Sr = 0.1194. ^87^Sr was corrected through the presence of Rb by monitoring the intensity of ^85^Rb and subtracting the intensity of ^87^Rb from the intensity of ^87^Sr using ^87^Rb/^85^Rb = 0.386 and a mass-bias correction factor determined from ^86^Sr/^88^Sr. The NBS 987 standard was analyzed at the start, then after every 6 enamel samples, and then at the end of the analytical session. The NBS 987 average ^87^Sr/^86^Sr analyzed together with the samples was 0.710244 (±0.00004; S5 Table).

### Freshwater strontium (^87^Sr/^86^Sr) and oxygen (*δ*^18^O) isotope analysis

Strontium isotope values were previously obtained from two archaeological animal bones (common opossum, *Didelphis marsupialis*, and lowland paca, *Cuniculus paca*) from archaeological deposits at CJD (^87^Sr/^86^Sr = 0.70703 and 0.70712; [103]). These species do not roam large distances and are currently present in the region today, so their values should reflect the local bioavailable strontium [104]. Strontium isotope values reported from other archaeological animal remains in central Panama were also included to reconstruct a baseline for the region (Table 2).

**Table 2:** Strontium isotope data from river water and archaeological animal remains.

Sample Number	Location Name/Archaeological Site	Sample Type	Species	^87^Sr/^86^Sr
1	Ladrones Cave	Enamel	Agouti (*Dasyprocta punctata*)	0.704268
2	Grande River (near El Caño/Sitio Conte)	Water	–	0.703901
3	Grande River	Water	–	0.703890
4	Chico River	Water	–	0.703978
5	Aguadulce Creek	Water	–	0.704988
6	Aguadulce Rockshelter	Bone	Gray fox (*Urocyon cinereoargenteus*)	0.705589
7	Santa María River (near Sitio Sierra)	Water	–	0.703858
8a	Sitio Sierra	Bone	Paca (*Cuniculus paca*)	0.705922
8b	Sitio Sierra	Bone	Paca (*Cuniculus paca*)	0.705475
9	Zapotal	Bone	Paca (*Cuniculus paca*)	0.707219
10	Vampiros Cave (coastal)	Bone	Cane mouse (cf. *Zygodontomys* sp.)	0.708954
11	La Villa River (near LS-15)	Water	–	0.704693
12	La Villa River (near LS-10)	Water	–	0.704718
13a	Cerro Juan Díaz	Bone	Opossum (*Didelphis marsupialis*)	0.707029
13b	Cerro Juan Díaz	Enamel	Paca (*Cuniculus paca*)	0.707122
13c	La Villa River (near Cerro Juan Díaz)	Water	–	0.704657
13d	La Villa River (near Cerro Juan Díaz)	Water	–	0.704734

**Fig 2:**
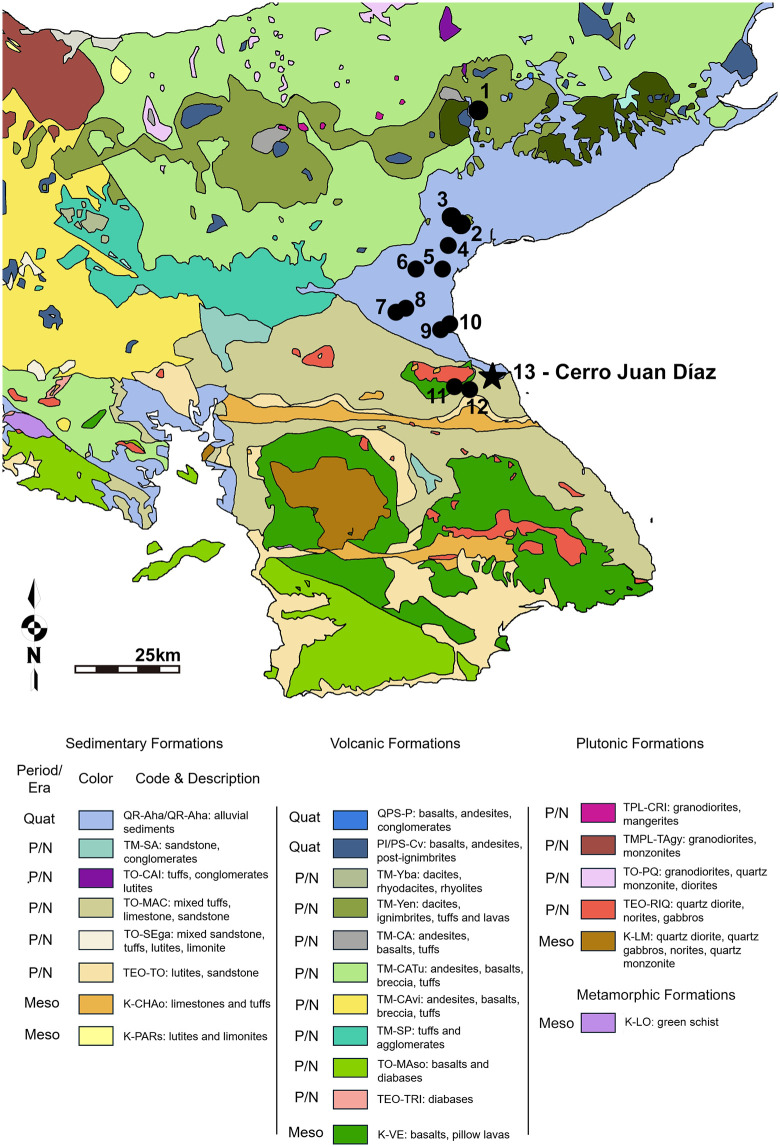
Geological map with reported strontium data for central Panama. Numbers correspond to locations on Table 2 where baseline strontium isotope data is available. Quat = Quaternary Period (2.6 million years ago to present), P/N = Paleogene/Neogene Periods (66 to 2.6 million years ago), Meso = Mesozoic Era (252 to 66 million years ago). Geological map republished from [109,110] based on a CC BY 4.0 license, with permission from Milton Solano, original copyright 2021. See these sources for details regarding geologic codes in Panama. Map created by Ashley Sharpe using Inkscape.

Sample numbers correspond with locations on Fig 2. Animal bone/teeth data previously reported in [103].

For the present study, two water samples were taken from the La Villa River near the CJD site. Using archaeological surveys of the region [19,20], we also obtained water samples near two other large (>80 ha) coeval sites upstream from CJD: Las Huertas (LS-10, ~ 5 km upstream) and La Chilonga (LS-15, ~ 14 km upstream). We also sampled the Santa María River near the site Sitio Sierra (30 km to the northwest), as it is another large river in the north Azuero Peninsula and Sitio Sierra has been a focus of previous isotopic investigations [103,105,106], and water from other rivers in the central Panama region (S7 Table). Oxygen isotopes were also tested from the water at these locations. Previously reported strontium isotopes from rivers in central Panama [107] are used for comparison in this study. While strontium isotopes from surface water can be subject to anthropogenic contamination and are influenced by the size and speed of water over different geological features [108], they are used here as an additional source of strontium data to support what is already known from the geology and the archaeological faunal strontium isotopes (Fig 2) [109,110].

River water samples for strontium and oxygen analysis were collected in sterilized 20 ml Nalgene HDPE bottles, and GPS points were taken of each location with a Garmin 64s recorder (see S7 Table). From each sample, 0.5 ml was tested with a Picarro L2120-I for *δ*^18^O and deuterium isotopes (*δ*D) in the University of Florida Light Stable Isotope Laboratory. Surface water *δ*^18^O has been previously examined across central Panama [111], but since *δ*D values have not, we do not use them to make interpretations in this study. However, we report them in S5 Table in case they could be of use in future studies. Results were standardized based on two internal University of Florida water standards (UW Antarctic water and Lake Tulane water) that were calibrated using international standards (USGS49 and USGS50). All isotope results are reported in standard delta notation relative to Vienna Standard Mean Ocean Water (VSMOW). Water *δ*^18^O values were also converted to VPDB in S5 Table using the following equation [112]:


$$\delta^{\text{18}}{\text{O}}_{\text{VPDB}}=\text{0}.\text{97002}*\delta^{\text{18}}{\text{O}}_{\text{VSMOW}}-\text{29}.\text{98}$$ (1)

Water oxygen isotope values in this study are intended to compliment the comprehensive assessment previously conducted across Panama by [111], in order to obtain values in close association with archaeological sites. As the La Villa River is the most accessible source of freshwater to the CJD site, it is most likely that people living around the hill or at nearby sites along the La Villa River would have been drinking from the river. The *δ*^18^O values in human enamel and water are not directly comparable, as tissue formation and fractionation from metabolism affect *δ*^18^O values (see [113] for a review of these and other factors involving oxygen isotopes). Furthermore, anthropogenic activities along rivers, including landscape changes along rivers, dams, and pollution, can affect the *δ*^18^O values of water today compared to the past, in addition to seasonal variation throughout the year in *δ*^18^O values that may differ today than in the past [113,114]. Nevertheless, when examined across a large region like central Panama, *δ*^18^O values from surface water are still valuable for understanding regional patterns which could help interpret *δ*^18^O values in human enamel.

For the strontium analysis, 10 ml of water was evaporated to dryness in pre-cleaned Teflon vials. The resultant residue was dissolved in 0.5 ml of 3.5 N HCl and rubidium (Rb) was separated from Sr on columns packed with AG50W-X12 resin. In short, the sample was loaded on the columns (15 cm long by 0.5 cm diameter resin volume) and the matrix was eluted with 17 ml of 3.5 N HCl. The Sr fraction was collected in 5 ml of 3.5 N HCl. The resultant Sr fraction for each sample was then evaporated on a hot plate to dryness, and dissolved in ~300 ųl of 3.5 N HNO_3_ and Sr was purified using the same ion chromatography procedure as was performed on the enamel samples, following [102]. Strontium isotope ratios were measured following the same method as the enamel samples on a Nu Plasma MC-ICP-MS. The NBS 987 average for the water was 0.710247 (±0.00001; S5 Table).

### Statistical analysis

Statistical software used in this analysis included Past (version 4.12), and RStudio (version 2022.02.3 + 492). Estimation of the proportion of dietary sources in human individuals was done using the multivariate model proposed by Froehle and colleagues [115], which accommodates both collagen (protein) and apatite (whole diet) components. Discriminate function analysis based on “carbon” and “nitrogen” components were determined using the following equations:


$$\text{F1}(\text{Carbon})=(\text{0}.\text{322}*\delta^{\text{13}}{\text{C}}_{\text{ap}})+(\text{0}.\text{727}*\delta^{\text{13}}{\text{C}}_{\text{col}})+(\text{0}.\text{219}*\delta^{\text{15}}\text{N})+\text{9}.\text{354}$$ (2)

$$\text{F2}(\text{Nitrogen})=(-\text{0}.\text{393}*\delta^{\text{13}}{\text{C}}_{\text{ap}})+(\text{0}.\text{133}*\delta^{\text{13}}{\text{C}}_{\text{col}})+(\text{0}.\text{622}*\delta^{\text{15}}\text{N})-\text{8}.\text{703}$$ (3)

The discriminate function method proposed by Froehle and colleagues [115] was based on isotopes from prior controlled animal feeding studies and populations of archaeological human remains reported by [116]; it was found to account for 98.8% of sample variance among five dietary clusters. Although studies have found that some dietary regimes may not adequately fit the model proposed by Froehle and colleagues [115], such as individuals with access to high-protein C_4_ plant species [117], it has proven a useful technique for the Central and South American societies where maize agriculture provided the dominant C_4_ dietary component [118–121].

To compare differences among sexes, age groups (adults and children), and time periods, we used the Mann-Whitney *U* test when comparing two groups, and the Kruskal-Wallace test for three groups. A Dunn’s post hoc test was done in cases where the Kruskal-Wallace test showed significant differences, in order to identify which groups were most different compared to one another. These tests were done because they are nonparametric and do not assume normality in the distribution of isotopic data. We did not include third molars in the tests, as there were too few to compare. In some cases, the number of samples compared from a group were small (below five samples), in which case the results of these tests should be treated with caution. The test results are interpreted taking into consideration other archaeological data from CJD and specific context of each buried individual.

To visualize and compare the isotopic differences in tooth enamel ^87^Sr/^86^Sr, *δ*^13^C, and *δ*^18^O between sex and age, a Principal Component Analysis was conducted using the *prcomp* function in the *stats* package from the R-core. These data were then grouped visually by sex with statistical ellipses around 40% of the data, and one “population” or community ellipse surrounding 95% of the data. To compare the variation in isotopic values among the groups, we used PCA1 and PCA2 values from the 95% ellipses to calculate the niche area of each group using the SIBER package in R [122]. The niche area represents a combination of dietary (carbon) and mobility (strontium and oxygen) information derived from the measured isotope values. We interpret that a difference in niche area between one or more groups indicates a difference in their dietary preference and/or mobility over time. We calculated the Bayesian bivariate mean (SEAb) and 95% credible intervals of each group using 95% of the data. Pairwise comparisons between groups were made by subtracting the posterior samples of one group from those of another (Δ = x_A − x_B). The resulting distribution was summarized by its mean, its 95% equal-tailed credible interval, and the probability that Δ is positive. Two groups were considered credibly different when the 95% interval did not include zero.

## Results

### Assessment of sample preservation

Of the 45 individuals tested for bone collagen and apatite samples, 39 had acceptable collagen yields (1% or greater of original bone sampled), %C, %N, and C:N. Two, Individuals 9 and 31, had unacceptable values for these quality checks, and so their bone collagen and apatite isotope values are not considered valid and were excluded from the results summary. Individual 26 had acceptable collagen yield and C:N but low %C and %N, so their bone collagen and apatite isotope values were also excluded from the results. Three individuals (Individuals 15, 20, and 27) had low collagen yields (0.4–1.0%), although acceptable C:N in all cases. While their isotope values should be considered with caution, we have included them with the analysis. Although Fourier-transform infrared spectroscopy (FTIR) was not performed as a quality check to validate the bone apatite integrity [123], we include bone apatite *δ*^13^C values in this assessment for those individuals where collagen values are considered valid. Interpretations of bone isotopes in this study focus on collagen *δ*^13^C and *δ*^15^N, rather than apatite *δ*^13^C.

The trace element concentrations from teeth were used to determine if the enamel samples had been subject to diagenesis. Comparison with previously published data of diagenetically altered samples by examining the concentrations of uranium, thorium, and rare earth elements ([101] and sources therein) shows that the enamel from CJD exhibits some elevation in these elements, but not enough to consider the samples diagenetically altered to the extent that the strontium isotopes are compromised (S6 Table).

### Variation in diet through time

**Fig 3:**
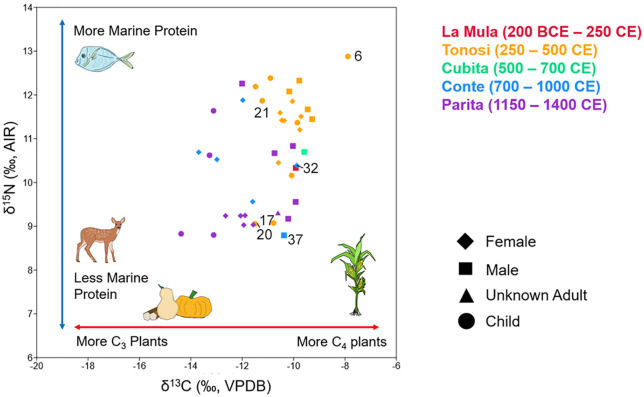
*δ*^13^C and *δ*^15^N isotope values from individuals at Cerro Juan Díaz. Samples from bone collagen, with individuals mentioned in text labeled.

**Fig 4:**
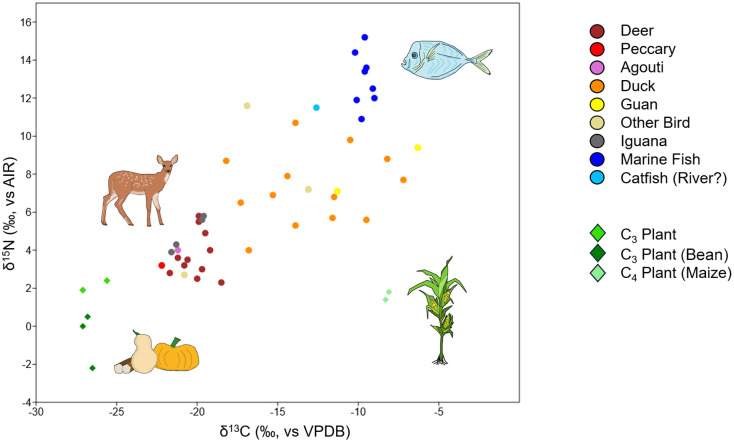
Archaeological faunal and modern plant *δ*^13^C and *δ*^15^N isotope values reported from previous studies in Panama and Costa Rica. Modern plant *δ*^13^C adjusted here by +1.5‰ to account for the Suess effect. Data from [66,105,124].

Comparison of the *δ*^13^C and *δ*^15^N values from bone collagen and *δ*^13^C values from bone apatite of 42 individuals shows that diets were variable over time at CJD (Fig 3; S3 Table). This variability appears to increase by at least 700 CE, during the start of the Conte ceramic style (700–1000 CE); before that time, diets among individuals were more consistent. A comparison of the human bone collagen *δ*^13^C and *δ*^15^N to reported isotope data from modern plants and archaeological fauna from southern Central America (Panama and Costa Rica) shows that the CJD individuals overlap particularly with fish (marine and freshwater) and birds, namely ducks (*Cairina moschata* or Muscovy ducks, possibly domesticated, and *Dendrocygna* sp.) and guans (*Penelope purpurascens*) (Fig 4) [66,105,124]. Several of the ducks and guans reported by Sugiyama and colleagues [66,124] came from different locations and chronological periods at CJD (S2 Table), and were interpreted to have been raised on maize given their elevated *δ*^13^C values. Thus, it is likely that a combination of fish, maize, and maize-fed birds in the human diets had elevated their *δ*^13^C bone collagen values above those of fauna that primarily consumed C_3_ plant species, such as deer and agoutis. Future baseline carbon and nitrogen isotope studies are warranted on flora and fauna in the central Panama region in order to more accurately model human diets (for example, as in [125] for Belize).

Comparison of the dietary proportion of different food groups (S2 Fig) shows that all individuals were consuming marine food and C_4_ plants, likely maize, although the proportion was generally greater for the Middle Ceramic individuals (200 BCE – 700 CE; at least 70% maize and 50% C_4_ protein, likely a marine component). One individual from the Conte style and eight from the Parita style (1150–1400 CE) exhibit a different dietary trend, where ≥65% of their protein comes from C_3_ sources and the proportion of maize has decreased. This pattern chronologically corresponds with the shift from C_4_ toward C_3_ plant consumption previously observed at the nearby village site of Sitio Sierra, 30 km to the northwest [103,105,106].

Diets among individuals of different ages and sexes overlap during the Middle Ceramic Period for bone collagen and apatite isotopes (S8 and S9 Tables). Adult males and children exhibit a slight but statistically significant difference in collagen *δ*^13^C values (Kruskal-Wallace *H* (*chi*^*2*^) = 6.022; *p* = 0.049). This is due to elevated *δ*^13^C_col_ values in adult males compared to children (adult male mean = −9.70‰; child mean = −10.46‰); adult females are between the two (mean = −10.21‰). Although the number of adult males with well-preserved bone collagen and apatite was too low to perform a statistical comparison with adult females (two males, five females), the isotope values of the two males overlap with the range of female values. However, during the Parita style, adult males buried at the site have significantly elevated *δ*^13^C_col_ values than children (*H* (*chi*^*2*^) = 10.68; *p* < 0.01; adult male mean = −10.22‰, child mean = −13.47‰). This difference is greater than that observed during the Middle Ceramic Period, for the mean difference between both adult males and children has moved from less than 1‰ to over 3‰. There is no statistical difference in any time period in *δ*^15^N among adult females, males, or children (S9 Table), indicating that the protein sources consumed by the three groups did not significantly vary within each period. Thus, the difference in *δ*^13^C_col_ among adult males and children during the Middle Ceramic and Parita styles appears to be primarily due to the amount of maize in the diet rather than consumption of marine resources (e.g., fish), as a difference in the latter should affect the nitrogen isotopes.

Outliers of *δ*^13^C_col_ and *δ*^15^N occur among some children. With the possible exception of Individual 46 (3–4 years of age), all children in this study are believed to be above the weaning age, so their nitrogen and carbon values should reflect a childhood diet rather than breastmilk that could otherwise raise the *δ*^15^N by ~1–3‰ and *δ*^13^C_col_ by ~1‰ [126,127]. Individual 6 has one of the highest *δ*^13^C_col_ (−7.9‰) and *δ*^15^N (12.9‰) values tested in any human in Panama to date (for comparison, see [103,105,106,128]. This child, between 5 and 6 years of age at death, was the only primary burial in a cylindrical pit feature containing at least 29 individuals arranged mainly in bundle burials and a commingled deposit comprising the occupants of a prior burial [70]. Individual 6 had been the last burial interred at this location near the southwest base of the hill. Unlike many individuals interred at CJD, this child did not have any noticeable pathologies, meaning they may have died from a sudden illness or other acute cause that did not leave signs on the skeleton. Their diet indicates that they consumed primarily maize and seafood, likely the high-trophic fish with elevated *δ*^15^N values that have been found at the site, including Pacific moonfish (*Selene peruviana*) and thread herring (*Opisthonema libertate*, [64]; see [129] for isotopic data for these species).

**Fig 5:**
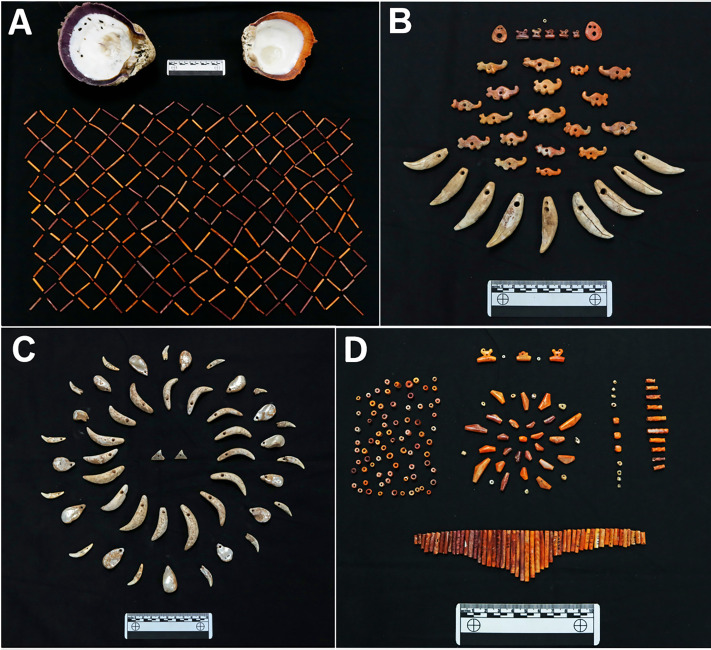
Marine shell and animal tooth ornaments interred with Tonosí style (250–500 CE) individuals examined in this study. (A) Thorny oyster (*Spondylus* sp.) shell tube beads interred with child Individual 20 (two modern shell valves included for comparison). (B) *Spondylus* shell ornaments and puma (*Puma concolor*) tooth pendants with child Individual 21. (C) Pearl oyster (*Pinctada mazatlanica*), carnivore teeth (mostly domestic dog, *Canis lupus familiaris*), and bull shark (*Carcharhinus leucas*) teeth pendants with tooth-like ornaments carved from a large marine gastropod, found with adult Individual 26. (D) *Spondylus* and pearl oyster beads found with child Individual 17. Photos by Ashley Sharpe.

Conversely, two other children from the same period, Individuals 17 and 20, appear to have been consuming less marine food and maize than their contemporaries (pre-700 CE). Individual 17 was a child of 6–8 years that exhibited signs of anemia and antemortem tooth loss due to prolonged illness or malnutrition and poor oral health, respectively. Accompanying this child were 133 carved thorny oyster (*Spondylus* sp.) bead ornaments (Fig 5). Individual 20, a child of 4–6 years, also had signs of anemia as well as extensive, unilateral dental calculus accumulation on the right deciduous molars. The *δ*^15^N values for these two children, both 9.1‰, were among the lowest for the individuals tested at CJD. Modern isotope studies of child and adult nutrition have shown that diet rather than disease is the primary cause of low *δ*^15^N values, usually from a lack of animal products (e.g., [130,131]; although see [132] for a review on the complexities of assessing anemia isotopically). The distinct isotope values for both children could indicate the consumption of a diet different from that of others buried at the site, which may also relate to the pathological lesions seen on both individuals, particularly those associated with anemia.

**Fig 6:**
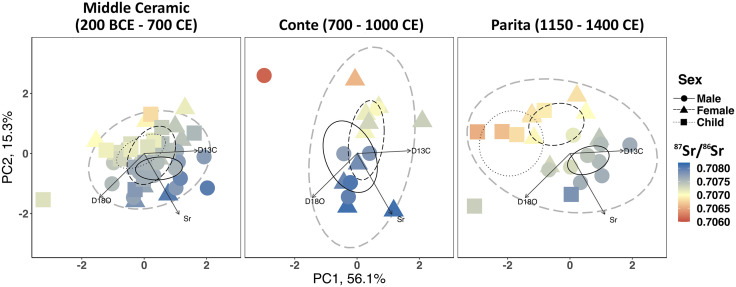
Principal component analysis (PCA) of the strontium, carbon, and oxygen isotopes from tooth enamel of individuals in the study. Statistical ellipses are drawn around 40% of the data for the demographic groups, and 95% of the data for the entire “population” (grey hashed line). Note how values become increasingly dispersed over time. See S3 Fig for a more detailed version of this data presented by tooth type.

Carbon isotopes from tooth enamel have been used in many studies in the world in order to understand human diet during tooth formation [118,133,134]. Diets from tooth enamel isotopes in this study (Fig 6; S3 Fig; S4 Table) generally support the patterns observed in the bone collagen and apatite data. The *δ*^13^C_en_ in individuals from the Middle Ceramic Period are elevated compared to individuals from later times, indicating that earlier individuals consumed more maize early in life than individuals who lived during the Conte and Parita components of the Late Ceramic Period. Furthermore, by comparing teeth that develop earlier and later in life, we see that individuals from the Middle Ceramic Period consumed greater proportions of maize over their lifetimes (*δ*^13^C_en_ values increase in 13 of 16 individuals with two or more teeth sampled). The opposite pattern occurs in individuals from the Conte group for both adult sexes (*δ*^13^C_en_ values of 5 of the 7 individuals with two teeth samples show a decrease with age) and the Parita group for all adult males but only one female. This suggests that after c. 700 CE, maize was more commonly consumed very early in life and increasingly less as children grew older.

The *δ*^13^C_en_ values in adult females, males, and children are similar in the Middle Ceramic Period (Kruskal-Wallace *H* (*chi*^*2*^) = 0.662, *p* = 0.72 for M1/deciduous teeth; *H* (*chi*^*2*^) = 2.606, *p* = 0.27 for older teeth). During the Conte period, adult females have slightly more elevated *δ*^13^C_en_ than adult males (female M1 mean = −4.54‰; older teeth mean = −5.01‰; male M1 mean = −5.88‰; older teeth mean = −7.17‰), whereas the trend is reversed during the Parita period (female M1 mean = −4.81‰; older teeth mean = −4.70‰; male M1 mean = −3.00‰; older teeth mean = −3.60‰). However, these values do not show a statistical difference. Interestingly and mirroring the bone collagen *δ*^13^C results, Parita adult males have higher *δ*^13^C_en_ values compared to children (Mann-Whitney *U* = 0, *z* = 1.945, *p* = 0.052 for M1/deciduous teeth; *U* = 1, *z* = 1.309, *p* = 0.190 for later teeth). For early teeth (M1/deciduous), this difference is almost statistically significant. Parita children have lower *δ*^13^C_en_ values than adult females as well, a pattern that is comparable with their *δ*^13^C_col_ values. The fact that Parita adult males have elevated *δ*^13^C_en_ values in both their earlier and later teeth, as well as their bone *δ*^13^C_col_, indicates they regularly consumed more maize than the adult females and children buried at the site throughout their life, even at a young age.

### Variation in mobility

Comparison of the strontium and oxygen isotope values in tooth enamel from different individuals shows that, like the diets, there is an increase in isotopic variability among individuals following 700 CE (Fig 6; S3 Fig; S8 and S9 Tables). This suggests that not everyone interred at CJD was originally from or lived near the site during life. Baseline ^87^Sr/^86^Sr and *δ*^18^O isotopes for CJD are difficult to measure exactly since the site is located near a geologically complex region of predominantly igneous rock and volcaniclastic sediments [107,135,136]. While the mountains of in Panama’s western half are actively volcanic, the La Villa River originates in the inactive western half of the Azuero Peninsula, which contains mixed Paleogene and Neogene igneous rocks, especially basalt (Fig 2) [135]. These would be expected to have low ^87^Sr/^86^Sr values (below 0.7060), but since the site is also near the ocean, it may have been influenced by marine strontium from sea spray (~0.7092) [137,138]. The eastern Azuero also contains some patches of sandstone and limestone (Fig 2, also observation by the authors who excavated at CJD). Other studies examining populations living in geologically complex areas near the coast, such as the Caribbean islands [139,140] and northern Europe [141–143], have found that while there is no exact means of determining what is “local” (i.e., someone born within ~5–10 km around a site), a consideration of various factors such as dietary input of terrestrial versus marine food based on faunal data and dietary isotopes can help identify the range of individuals who may be “local”. Oxygen isotope data, primarily influenced by the *δ*^18^O in drinking water in humans, can help distinguish local and non-local individuals [113,144].

Strontium isotopes from the faunal samples show that the two animals had similar strontium values (0.70703 and 0.70712), which were elevated compared to the values obtained from the La Villa water samples near the site (Table 2) [103]. All four water samples from the La Villa River had similar ^87^Sr/^86^Sr values (0.70466–0.70473), matching a value obtained from a previous geochemical survey of the river (0.70462) [107]. They also had similar, low strontium concentrations (average 0.110 ppm; ocean water is ~ 8 ppm) indicating that saline water from the mangroves near CJD was not concentrated enough to influence strontium isotope values from drinking water at CJD.

The ^87^Sr/^86^Sr values among the humans buried at CJD average 0.70740, with a range of 0.70612 to 0.70811. No individual has a ^87^Sr/^86^Sr value as high as the expected sea water value (~0.7092), but none match the river water ^87^Sr/^86^Sr near the site, rather falling into a range in between and overlapping with the average of the two animals tested from the site (0.7071). The lowest variability among ^87^Sr/^86^Sr values in individuals is observed during the time before 700 CE (^87^Sr/^86^Sr = 0.7068–0.7080; Fig 6). Conte individuals exhibit the greatest variability (^87^Sr/^86^Sr = 0.7061–0.7081). Comparison of dental enamel Sr concentrations with ^87^Sr/^86^Sr values does not reveal a statistically significant correlation (Pearson’s correlation *r* = 0.3543; S4 Fig). Similarly, there is little correlation between δ^15^N and ^87^Sr/^86^Sr (Pearson’s correlation *r* = 0.3542; S5 Fig), meaning that the proportionally greater marine diet exhibited among some individuals did not appear to significantly alter their ^87^Sr/^86^Sr value. As the dietary isotopes showed, all individuals at CJD were eating marine food to varying extents, so their ^87^Sr/^86^Sr values were all elevated above that of drinking water and terrestrial food sources. A more comprehensive assessment of ^87^Sr/^86^Sr in animals and plants in the CJD and Parita Bay area is warranted in order to understand how sea spray influences regionally bioavailable strontium.

There is no statistical difference in ^87^Sr/^86^Sr among sexes during the Middle Ceramic Period or Conte style (Mann-Whitney *U* = 60, *z* = 1.552, *p* = 0.12; *U* = 29, *z* = 0.054, *p* = 0.96, respectively; see S9 Table for comparisons of specific teeth). There is, however, a significant difference between Middle Ceramic Period adult males and children in the first molar (*U* = 12, *z* = 2.201, *p* = 0.03), but not adult females and children (*U* = 20, *z* = 0.424, *p* = 0.67), nor between adult females and males (*U* = 4, *z* = 1.599, *p* = 0.11). We were unable to test teeth from Conte children since they did not meet our sampling requirements (see Methods). Adult females and children from the Middle Ceramic Period and Parita style generally have lower and more variable ^87^Sr/^86^Sr values than the males (Fig 6; S8 Table).

During the Conte style, five individuals show a decrease in ^87^Sr/^86^Sr in their second tooth. The two most significant of these include Individual 32, an adult female (0.70713 to 0.70659) and Individual 37, an adult male (0.70705 to 0.70612). These two individuals showed a decrease in *δ*^13^C_en_ between earlier and later teeth as well, and Individual 37 has the lowest *δ*^15^N of any adult male tested at the site, meaning he may have consumed less marine food than the others. Significantly, these two individuals were interred as “bundle burials”—a secondary burial mode in which the bones of the deceased were removed from their original place of deposition and specially wrapped in a package before final deposition [75]. It would appear that these individuals moved during their early childhood to another location with lower ^87^Sr/^86^Sr and less dependence on maize agriculture and marine food, likely further inland or upstream from CJD. Given their secondary burial mode, it is possible that the two were originally interred or their bodies maintained at another location and transported for final burial at CJD sometime later.

Most drinking water from CJD would likely have come from the river, and so human enamel *δ*^18^O should reflect similar regional trends in values (see S7 Table for SMOW and VPDB values). The *δ*^18^O values reported from water in this study reflect those reported in [111] from the Pacific coast of Panama (*δ*^18^O_VSMOW_ ~ −6‰ to −10‰; this study: −6.5‰ to −8.0‰). According to Lachniet and colleagues [111], *δ*^18^O_VSMOW_ values of surface water closer to the Caribbean side of the isthmus (<50 km from the coast) tend to be −6‰ or higher, due to greater rainfall on the Caribbean side and a decrease past the central mountains toward the Pacific slope. This is particularly the case for the eastern Azuero Peninsula where CJD is located, which receives the least annual rainfall in Panama. The *δ*^18^O_VPDB_ values reported from Holocene stalagmites also reflect this trend [111], with Pacific slope values clustering around −8‰ and Caribbean slope values around −4‰.

Human enamel *δ*^18^O values in the present study are reported against the VPBD standard, and ranged from −5.21‰ to −7.19‰ (S8 Table). First molars and deciduous teeth may show ~1‰ enrichment in *δ*^18^O from breastfeeding [113,145], and so are treated separately in this study from other teeth. As well, deciduous teeth and first molars can have ^87^Sr/^86^Sr values reflective of the mother, and so should be treated with caution. Comparing enamel *δ*^18^O values to those of ^87^Sr/^86^Sr, we find further evidence that individuals moved (or were moved post-mortem) to the site from other locations. Adult females and males do not differ significantly in the Middle Ceramic Period (S8 and S9 Tables), although females have a slightly broader *δ*^18^O_en_ range as they did with ^87^Sr/^86^Sr (Fig 6; S3 Fig). Children *δ*^18^O_en_ values do not differ significantly compared to adults during this period, with the exception of Individual 20, who had an unusually high *δ*^18^O_en_ value of −5.2‰. This individual is one of the two children with unusually low *δ*^15^N during the Middle Ceramic Period. The elevated *δ*^18^O_en_ value is more similar to values found in the water from northern Panama [111], as surface water *δ*^18^O values generally increase toward the Caribbean. The *δ*^18^O_en_ values among adult individuals during the Conte style overlap between sexes (Mann-Whitney *U* = 3, *z* = 0.884, *p* = 0.38 for first molars), as they had done for ^87^Sr/^86^Sr. During the Parita style, adult male and female *δ*^18^O_en_ values overlap more than child *δ*^18^O_en_ values for both early (M1/deciduous) and later (canine, premolar, and M2) teeth as the child *δ*^18^O_en_ values are slightly higher, although the difference between children and adult *δ*^18^O_en_ values is not significant. Three of the elevated Parita children *δ*^18^O_en_ values resemble the outlier child, Individual 20, in the Middle Ceramic Period, with values exceeding −6.0‰. It is unlikely that this is due to breastfeeding, since similar values would be expected from the first molars of the adults in the population as well (reflecting the values of those adults as infants). Due to the limited number of samples per time period in this study, oxygen isotope outliers should be treated with caution [144].

### Overall niche variation

Combining the diet and mobility isotope values (carbon, strontium and oxygen) from tooth enamel supports the conclusions that groups became more variable after 700 CE (Fig 6; S3 Fig; S8, S9, and S10 Tables). PCA analysis shows that the variation (i.e., ellipse area) of the populations is greater after 700 CE, and Bayesian analysis of SEAb supports this (S6 Fig). SEAb of the Middle Ceramic Period, Conte style, and Parita style were 13.71, 30.17, and 24.80 PC units, respectively. The posterior mean difference between the Middle Ceramic and Conte period 95% ellipses (variation) was −16.72 PC units (95% equal-tailed credible interval: −32.91 to −2.00); P(Δ > 0) = 0.01, meaning the Middle Ceramic variation is credibly smaller than the Conte variation. The posterior mean difference between the Middle Ceramic and Parita period variation was −11.68 PC units (95% equal-tailed credible interval: −28.61 to −3.30); P(Δ > 0) = 0.01, meaning the Middle Ceramic variation is also credibly smaller than the Conte variation. Finally, the posterior mean difference between the Conte and Parita period variation was 6.05 PC units (95% equal-tailed credible interval: −9.30 to 25.50); P(Δ > 0) = 0.76. As the interval crosses zero, there is little evidence that the variation of the Conte and Parita individuals are different.

## Discussion

The results of the isotope analysis at CJD reveal a dynamic community that changed and evolved over time, likely in part due to social developments that occurred throughout central Panama and the broader Isthmo-Colombian Area as chiefdoms coalesced into more regimented hierarchies and growing populations necessitated a broader variation in occupational specialties and activities among individuals. While diets and movements appear relatively consistent among individuals interred at CJD until 700 CE, significant variation among individuals based on age at death and sex occur thereafter. These isotopic data can be explained from what is known from the socioeconomic history of central Panama in the 1500 years prior to Spanish contact.

Before the transition of the Middle to Late Ceramic periods around 700 CE, archaeological evidence from CJD and central Panama as a whole suggests that settlements, although likely directed by a chief or individual(s) of similar standing, were mostly egalitarian in terms of everyday activities and distribution of resources [9,14,57]. The isotopic data in this study show that most people ate a diet focused on marine resources and maize agriculture. Burials were often communal, and, as occurred at CJD, interments were often performed in the same location over hundreds of years, regardless of sex or age at death. Preliminary biodistance analysis suggested that these communal burials were not restricted to specific family units, and that adults buried at CJD at this time were more phenotypically similar to each other, regardless of sex, than they were to non-adults [146].

Significant dietary variation during the Middle Ceramic Period occurred among children, the three outliers being unique burials with many special offerings (Individuals 6, 17, and 20; Fig 5). The practice of burying special items, including shell ornaments and gold, with children more frequently than with adults during this period has been reported from archaeological sites throughout central Panama, signaling that an individual’s age was more important than social status in decisions on individual mortuary offerings [57,147]. This pattern appears to be unique regionally, considering the propensity of sumptuary artifacts as status markers included in mostly adult burials in the surrounding Mesoamerican and Andean areas. Published examples of this practice in Panama include children with gold artifacts at Playa Venado [32], a cemetery site coeval to that of CJD’s Cubitá and Conte period of occupation, and El Indio in the southern Azuero Peninsula [17,54,57,147]. Stone and shell beads were found almost exclusively in infant and child burials at Sitio Sierra and Cerro Mangote, along the Santa María River north of CJD [148,149]. Currently, the oldest metal artifact recovered in Panama is a copper ring associated with a child buried at CJD (Individual 21). Other diverse items were also associated with this child, including several drilled *Spondylus* shell beads and carnivore teeth, among them puma (*Puma concolor*) and ocelot (*Leopardus pardalis*; Fig 5B) [26,150].

Coastal-dependent communities such as CJD are often difficult to assess in terms of ^87^Sr/^86^Sr and mobility, for the ^87^Sr/^86^Sr of marine resources, including salt, will alter human isotope values [139,141,143]. At CJD where the local geology is largely volcanic sediment (Fig 2), individuals should have strontium isotope values matching that of the local fauna (~0.7071); however, the site’s proximity to the coast and obvious dependency on marine resources entails that human ^87^Sr/^86^Sr values will be higher, closer to the marine ^87^Sr/^86^Sr of 0.7092 from sea spray [151]. It is therefore impossible to predict what the exact ^87^Sr/^86^Sr value of a “local” inhabitant living in the proximity of the CJD hill would have been, for individuals may have varied depending on their consumption of marine food and salt. However, we can conclude that the local faunal ^87^Sr/^86^Sr values provide a lower baseline for the bioavailable strontium, with the sea water ^87^Sr/^86^Sr value being an upper limit.

Differences in ^87^Sr/^86^Sr and *δ*^18^O_en_ among adult females and males at CJD prior to 700 CE indicate that members of one sex may have spent their early childhood years at another location, although perhaps not far from CJD since there are no outliers. Assuming most adults consumed similar quantities of marine food based on their similar *δ*^15^N values at this time, the broader variation in ^87^Sr/^86^Sr in adult female teeth may indicate some individuals were born at another location and moved as children or adolescents to CJD. Currently and historically, versions of both patrilocal and matrilocal postmarital residence systems occur in Panama. The Ngäbe and Buglé in western Panama are generally patrilocal [152,153], whereas the Guna who live along Panama’s northeast coast have generally practiced a matrilocal system [154,155]. Matrilocality is also practiced by the Bribri and Naso Tjerdí of Western Panama [152,156,157]. The Emberá and Wounaan in eastern Panama practice both postmarital residence systems [158–160].

The significant difference between children and male ^87^Sr/^86^Sr values in first molar/deciduous teeth during the Middle Ceramic Period is also of interest. The variation is partly driven by low ^87^Sr/^86^Sr in children who were already noted to have unusual values for other isotopes, such as Individual 6, who had the highest *δ*^15^N and *δ*^13^C_col_ values in this study, and Individual 20, who had the highest *δ*^18^O_en_ value in this study and one of the lowest *δ*^15^N values. This suggests that these children were not from CJD originally, but perhaps had come from another location. The burial offerings found with these Middle Ceramic Period children, such as the *Spondylus* shell beads, may have had something to do with why they were buried at CJD specifically and not their place of origin.

Dietary and mobility patterns significantly changed at CJD following 700 CE, perhaps reflective of broader changes in regional sociocultural dynamics and interaction spheres around this time. From about 200–800 CE, the geographical bounds of material culture goods containing shared designs characteristic of the Gran Coclé Semiotic Tradition increasingly expanded eastwards beyond central Panama to include most of the Pacific coast and islands of the Gulf of Panama, indicating either the movement of artisans or their goods, as well as a widespread network of exchange [29–31]. Following 500 CE, decorated Cubitá and Conte wares appear to have been exchanged as far as the Darien and Pearl Islands to the east [31,32,161,162], and the Gulf of Chiriquí and the Coiba Archipelago to the West [11,163]. During the Conte style, extensive elite tombs were established at the Gran Coclé mortuary centers of El Caño and Sitio Conte, containing thousands of fine-crafted objects of gold, painted ceramic, precious stones, and other materials [51,58–60]. By about 1000 CE, however, the geographic bounds of this cultural continuity had diminished, with the easternmost bounds archaeologically identified in the area of the El Valle volcano—approximating ethnolinguistic frontiers observed by the Spanish at their arrival to the Pacific coast in the early 16^th^ century [32,76,161,164].

The isotope data from individuals buried at CJD who lived during the Conte style shows that diets became highly variable (Fig 3), and maize was not consumed to the same extent as it had been centuries earlier. It is unlikely that this shift was due to decline in marine resources in the diet, since archaeofaunal evidence for fish persist at the site throughout its history [43,64], and some individuals maintain high *δ*^15^N values, such as Individual 27 (*δ*^15^N = 12.3‰). Although the collagen preservation for Individual 26 did not meet the requirements for this study, this adult male was buried with an unusual number of grave items for this period, including mother-of-pearl pendants (*Pinctada mazatlanica*) and bull shark teeth (*Carcharhinus leucas*; Fig 5C); the strontium values for his enamel samples suggest he did not move during life, and likely lived near the CJD site. The two individuals who consumed the greatest quantities of maize during this time, Individuals 32 and 37, apparently moved away from the coast during childhood according to the strontium isotopes in their teeth, perhaps moving to an inland location in the Azuero Peninsula. It is unclear whether these individuals returned to CJD later in life or only after death, since these individuals were interred as secondary burial bundles. It may be that the individuals in the Conte bundles were buried at CJD because it was a well-known and socially significant cemetery near their original place of residence by the coast. Such post-mortem transfer back to family or clan cemeteries for secondary burial has been noted as a key element of continued interactions between the living and the dead among many surviving indigenous groups throughout the Isthmo-Colombian Area, including among the Bribri of Panama [156,165].

Dietary and mobility patterns become increasingly variable during the Parita style, as ceramic and metal artifacts continued to be shared and distributed throughout central Panama [29–31]. Studies of settlement patterns across the Gran Coclé region show that population centers shifted during this time [17,19,20], although the area immediately around CJD remained continuously occupied. This may explain the disparity in diets among individuals at CJD, as a wide range of social occupations and roles may have coexisted, and the La Villa and neighboring river valleys would have numerous settlements engaged in trade and kinship alliances, as well as occasionally conflict and warfare. Adult females and males appear to have been consuming different diets, with males eating more maize and females consuming diets resembling those of children (Fig 3). This isotopic pattern has also been identified at Sitio Sierra to the north [103,105], which was a smaller settlement than CJD at the time (see S2 Fig for comparison between both sites). The pattern suggests that adult males were eating separate diets compared to adult females and children, possibly because they were hunting, fishing, or working in agricultural areas and brought with them a portable maize gruel to drink (perhaps fermented “chicha”) or bread to eat. Adult females may have been engaged in some of these activities as well, as is apparent from the variability in the diets of some individuals, but the majority were consuming less maize. The fact that first molars among CJD adult males also showed greater maize consumption than females indicates that this dietary difference began at infancy during the Parita style.

Interestingly, the overall dependency on maize appears to decline from the Middle Ceramic Period to the Parita style of the Late Ceramic Period. This same trend has been observed at Sitio Sierra, where the earlier occupation (50 BCE – 400 CE) had significantly higher *δ*^13^C_col_ than the later occupation (950–1150 CE), showing almost no overlap [103,105]. It is unlikely that this difference was due to a change in dependency on marine food, as the human bone nitrogen values overlapped in both occupations, and marine and freshwater fish were important throughout the site’s history [166]. This suggests that individuals in central Panama consumed increasingly more C_3_ agricultural plants, perhaps manioc (*Manihot esculenta*), squash (*Cucurbita* sp.), and a variety of fruit crops [37,167]. The shift away from maize as the primary agricultural staple in Panama is intriguing, since isotopic studies have shown that maize remained dominant in diets across Mesoamerica to the north until and even after the arrival of Europeans [134,168–170]. Although carbonized maize grains have been found at CJD dating to all periods in the present study [167], and a microbotanical test for starch identified maize on one metate [171], a thorough archaeobotanical study has yet to be done at the site. Such a study could determine which C_3_ plants had been consumed.

Mobility patterns also became increasingly variable among individuals during the Parita style, which would in part explain the variation in diets. There is more variation in ^87^Sr/^86^Sr and *δ*^18^O_en_ among females and children at this time, although there are more *δ*^18^O_en_ outliers in children than adult females. This suggests that at least some children buried at CJD during the Parita style were not from the site; their lower ^87^Sr/^86^Sr and higher *δ*^18^O_en_ values suggest they came from a region to the north or more inland (Fig 2) [111]. There is evidence of non-adult individuals buried at other hill sites near the coast around this time in Panama: multi-isotope analysis of an adolescent female buried at Cerro Brujo in northwest Panama c. 1300 CE showed that she likely had not lived at the site nor the coast during life, and her burial post-dated the site’s abandonment by over a century [103,172]. Parita burials at CJD and the coeval burial Cerro Brujo may be evidence of a mortuary practice or shared belief system across regional boundaries in the Isthmo-Colombian Area whereby individuals were interred at a sacred elevated point on the landscape near the ocean. Similar burial practices continue today among several indigenous groups throughout the region [173–175].

## Conclusions

Results from this isotopic assessment of 49 individuals from CJD, one of the largest sites excavated in Panama, show that dietary patterns and movements among individuals changed significantly around 700 CE. While we cannot determine the social ranks of individuals recovered from CJD, we can see that dietary practices were similar among individuals of all ages and both sexes before 700 CE, and thereafter became more variable. Adult females appear to have moved more frequently than adult males, possibly due to marital patterns necessitating females move to the residence of or near her partner’s family. If this is the reason for the broader isotopic ranges in females, it would appear that the practice of moving to the household of the husband occurred as far back as two millennia ago; strontium and oxygen isotope analysis of individuals prior to this date is largely lacking, and would be interesting to compare to see if similar marital and kinship practices occurred even earlier.

The treatment of children who died before adulthood is of particular interest, for it is unique in central Panama compared to many other regions of the world. At CJD and other Gran Coclé sites, children are often the recipients of special burial goods, particularly shell and metal ornaments. The isotope analysis in this study has shown that some children had unique diets, perhaps related to why they had died young. Furthermore, some children in the Middle Ceramic and Parita periods do not appear to have been local to the CJD area, implying they were buried there for a specific purpose. It is difficult to interpret such patterns for a society that lacked written records, although it is likely that CJD’s long history as a cemetery site may have been part of the reason children were buried there.

As the river valleys throughout the Parita Bay region contained numerous closely-spaced settlements [14,19–21], a more thorough regional assessment of the strontium and oxygen isotope variability is needed in order to determine the range and nature of mobility among individuals in the area. Although CJD was more than a cemetery, the central hill may have been an iconic location for interring the dead. More strontium and oxygen isotopic data is needed from the flora and fauna in the region in order to better define the range of isotopes that would be expected among humans living around the lower La Villa River valley, as well as the neighboring rivers.

While archaeologists have paid considerably more attention to the Mesoamerica and Andean cultures to the north and south of Panama, the people of the central isthmus that connected two continents had a fascinating and culturally distinct society that stands out on its own. Tracking the isotopic history of individuals at a single site across 1500 years, this study shows that Gran Coclé society changed over time, shifting from an egalitarian community where diets and mobility trends were generally uniform to a social organization where diets and movements were highly variable among individuals, and often variable according to one’s age and sex. Ongoing archaeological research in the isthmus will undoubtedly provide answers to some of the intriguing patterns revealed in this study.

## Supporting information

S1 Fig Map of the Cerro Juan Díaz site and locations of Operations 3, 4, and 5, from where human individuals in this study were found. Illustrations by Claudia Díaz, Benoit Desjardins, and Luis Sánchez.
(TIF)

S2 Fig Comparison of diets using isotopes from individuals at Cerro Juan Díaz and Sitio Sierra. (A) *δ*^13^C of apatite and collagen compared to dietary feeding studies [115], from Cerro Juan Díaz and Sitio Sierra (light gray points: 50 BCE – 400 CE; black points: 950–1150 CE); (B) dietary discriminant function analysis compared to feeding studies [115]. Data from Sitio Sierra reported from [103].
(TIF)

S3 Fig Strontium, oxygen, and carbon isotope values from tooth enamel reported by tooth type (first molar, second molar/premolar, third molar). For the ^87^Sr/^86^Sr graphs, the blue line designates the sea water isotope value (0.7092), the yellow line designates the average of small animal bones tested from the site (0.7071), and the pink line designates the average of water samples taken near CJD (0.7047). Note that most individuals had only two and not all three teeth sampled (see S4 Table for complete list).
(TIF)

S4 Fig Comparison of enamel strontium concentrations (parts per million, ppm) and ^87^Sr/^86^Sr in humans from CJD. There is a weak linear regression between the two variables (Pearson’s correlation *r* = 0.3543).
(TIF)

S5 Fig Comparison of enamel ^87^Sr/^86^Sr and δ^15^N_col_ in humans from CJD. There is a weak linear regression between the two variables. Assuming higher nitrogen isotope values indicate more marine protein in the diet, these results indicate that strontium isotope values are not significantly influenced by marine protein at CJD (Pearson’s correlation *r* = 0.3542).
(TIF)

S6 Fig SIBER (Stable Isotope Bayesian Ellipses in R) density plot showing Bayesian Standard Ellipse Areas (SEAb). Box plots are the Bayesian results from 20,000 model iterations after 1,000 were thinned. Points represent Bayesian modes, red crosses are means, and the boxes represent the 50%, 75%, and 95% credible intervals, respectively.
(TIF)

S1 Table Ceramic chronology of central Panama used in this study. For a more detailed review of these temporal periods, see [31] and [89].
(XLSX)

S2 Table Carbon (*δ*^13^C) and nitrogen (*δ*^15^N) isotope data from bone collagen from archaeological fauna and modern plants in the isthmus area, including Costa Rica and Panama. Norr’s [105] modern plant *δ*^13^C values have been adjusted here by +1.5‰ to account for the Suess effect [176], as she specified that her reported values were not corrected for the difference between preindustrial and modern decrease in ^13^CO_2_ (vs ^12^CO_2_) due to the burning of fossil fuels.
(XLSX)

S3 Table Bone collagen and apatite isotopes from individuals at CJD. ^14^C dates calibrated with IntCal20. Samples marked in yellow were reported previously in [103].
(XLSX)

S4 Table Enamel isotopes from individuals at CJD. ^14^C dates calibrated with IntCal20. Samples marked in yellow were reported previously in [103].
(XLSX)

S5 Table Information regarding the laboratory standards used in this study. (XLSX)

S6 Table Trace element concentrations from tooth enamel, in ppm. Samples marked in yellow were reported previously in [103].
(XLSX)

S7 Table GPS coordinates and isotopes from river water samples in central Panama. Month and season recorded in case this may influence oxygen and deuterium isotope values. Oxygen isotope vales converted from VSMOW to VPDB using the equation proposed by [112].
(XLSX)

S8 Table General statistics for enamel and bone isotopes of humans from CJD. (XLSX)

S9 Table Statistical tests comparing variance in sample groups. M3 (“wisdom”) teeth excluded due to low number of samples. Each statistical test only includes one sample per individual. Significant differences (*p* < 0.05) demarcated in red. For significant values, see Dunn’s post hoc analysis at bottom of tables.
(XLSX)

S10 Table Calculation of SIBER analysis between PCA1 and PCA2. (XLSX)
