# Supplementary figures and images for: Isotopic evidence of increased societal diversification in Pre-Columbian Panama
Source: PLoS One. 2025 Nov 6;20(11):e0335678. doi: 10.1371/journal.pone.0335678 (PMC12591407; doi:10.1371/journal.pone.0335678)

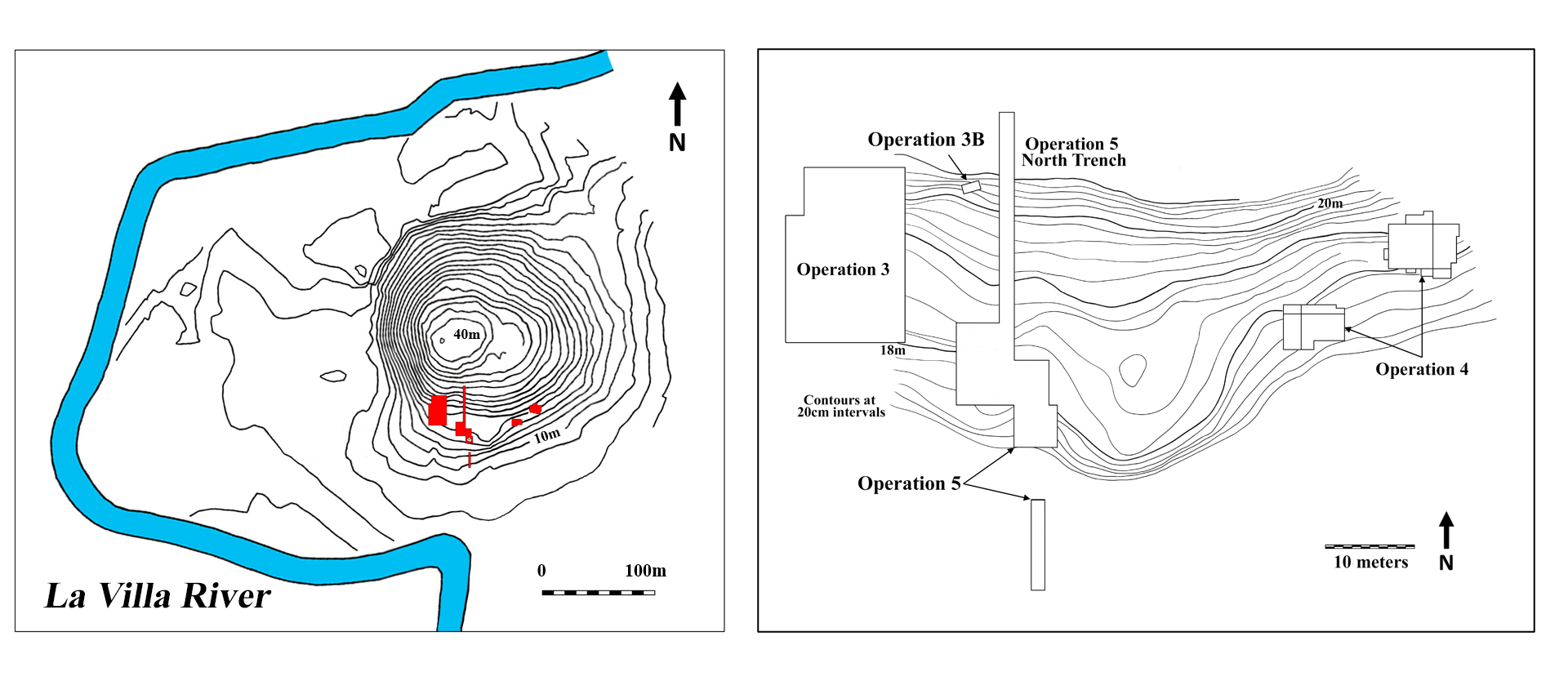

Supplement: S1 Fig — Illustrations by Claudia Díaz, Benoit Desjardins, and Luis Sánchez. (TIF) [file pone.0335678.s001.tif]

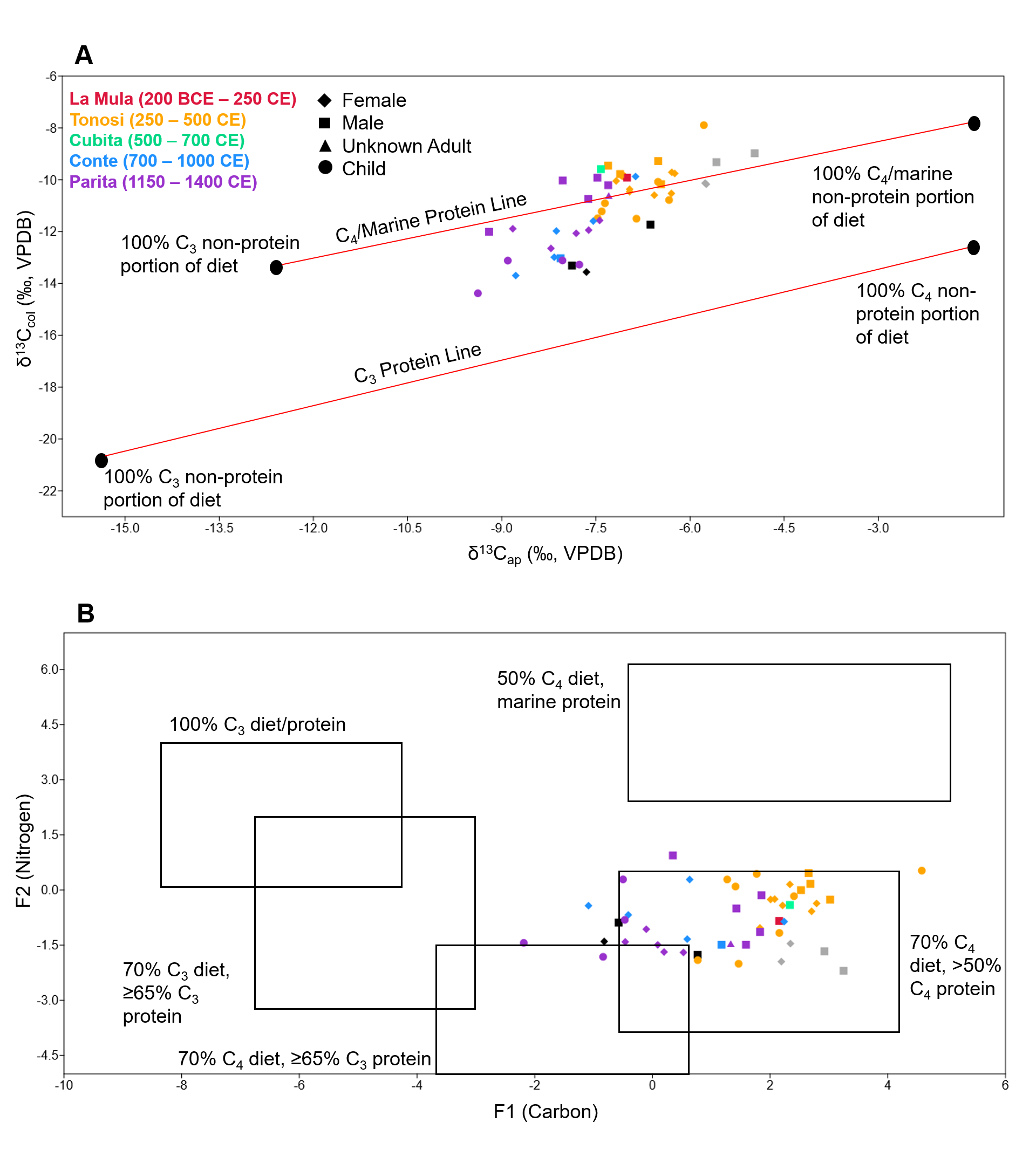

Supplement: S2 Fig — (A) δ13C of apatite and collagen compared to dietary feeding studies [115], from Cerro Juan Díaz and Sitio Sierra (light gray points: 50 BCE – 400 CE; black points: 950–1150 CE); (B) dietary discriminant function analysis compared to feeding studies [115]. Data from Sitio Sierra reported from [103]. (TIF) [file pone.0335678.s002.tif]

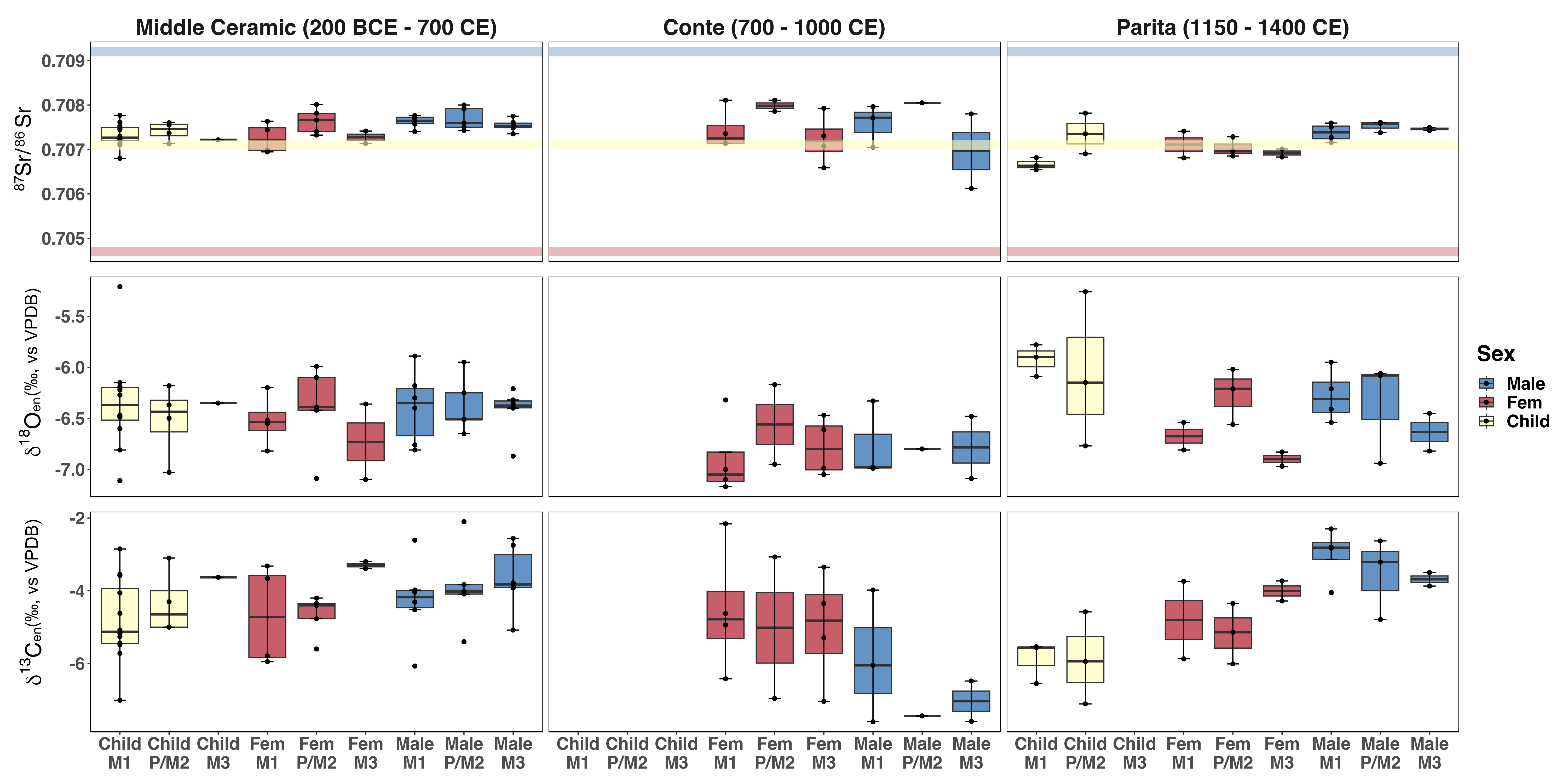

Supplement: S3 Fig — For the 87Sr/86Sr graphs, the blue line designates the sea water isotope value (0.7092), the yellow line designates the average of small animal bones tested from the site (0.7071), and the pink line designates the average of water samples taken near CJD (0.7047). Note that most individuals had only two and not all three teeth sampled (see S4 Table for complete list). (TIF) [file pone.0335678.s003.tif]

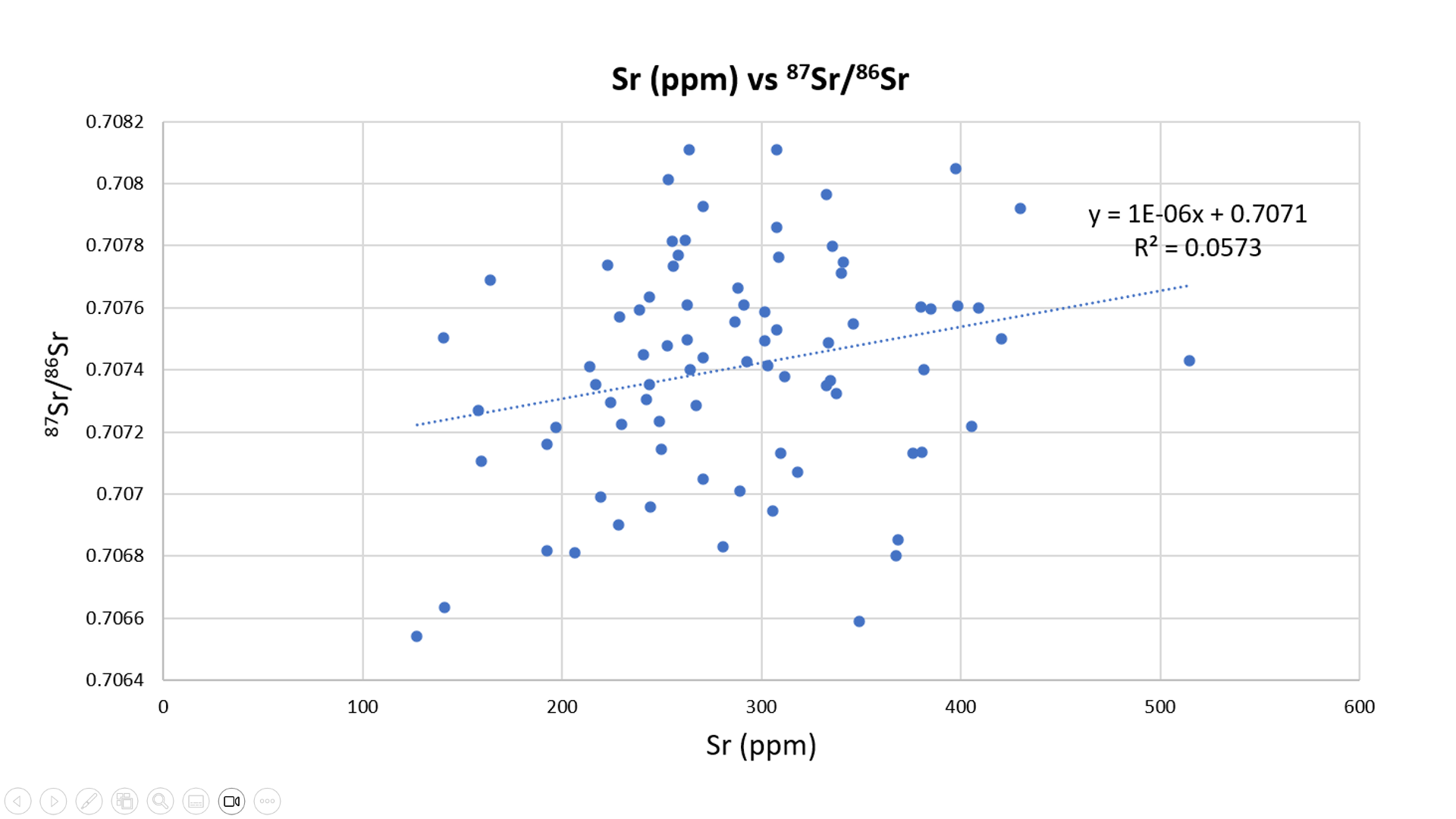

Supplement: S4 Fig — There is a weak linear regression between the two variables (Pearson’s correlation r = 0.3543). (TIF) [file pone.0335678.s004.tif]

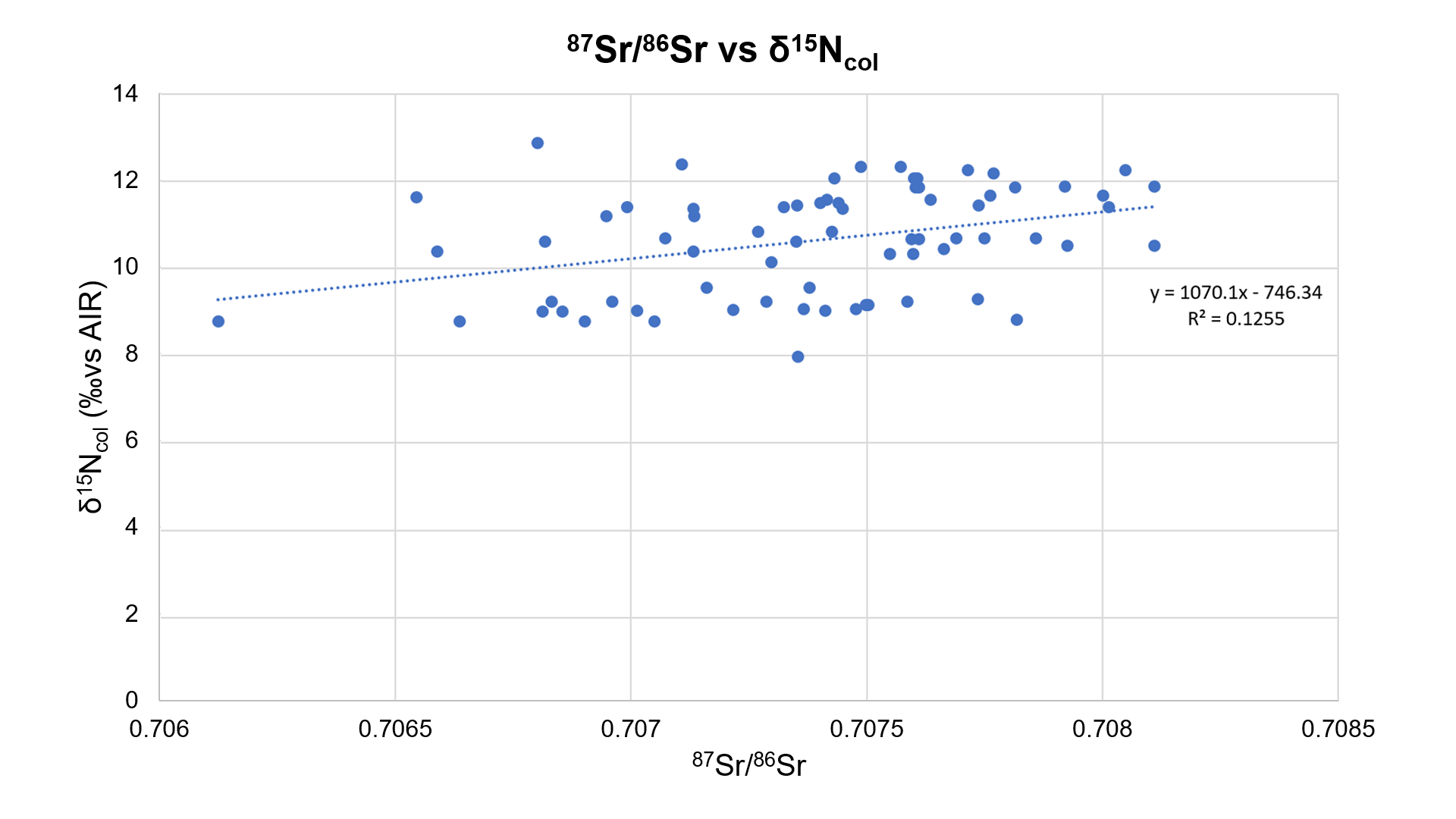

Supplement: S5 Fig — There is a weak linear regression between the two variables. Assuming higher nitrogen isotope values indicate more marine protein in the diet, these results indicate that strontium isotope values are not significantly influenced by marine protein at CJD (Pearson’s correlation r = 0.3542). (TIF) [file pone.0335678.s005.tif]

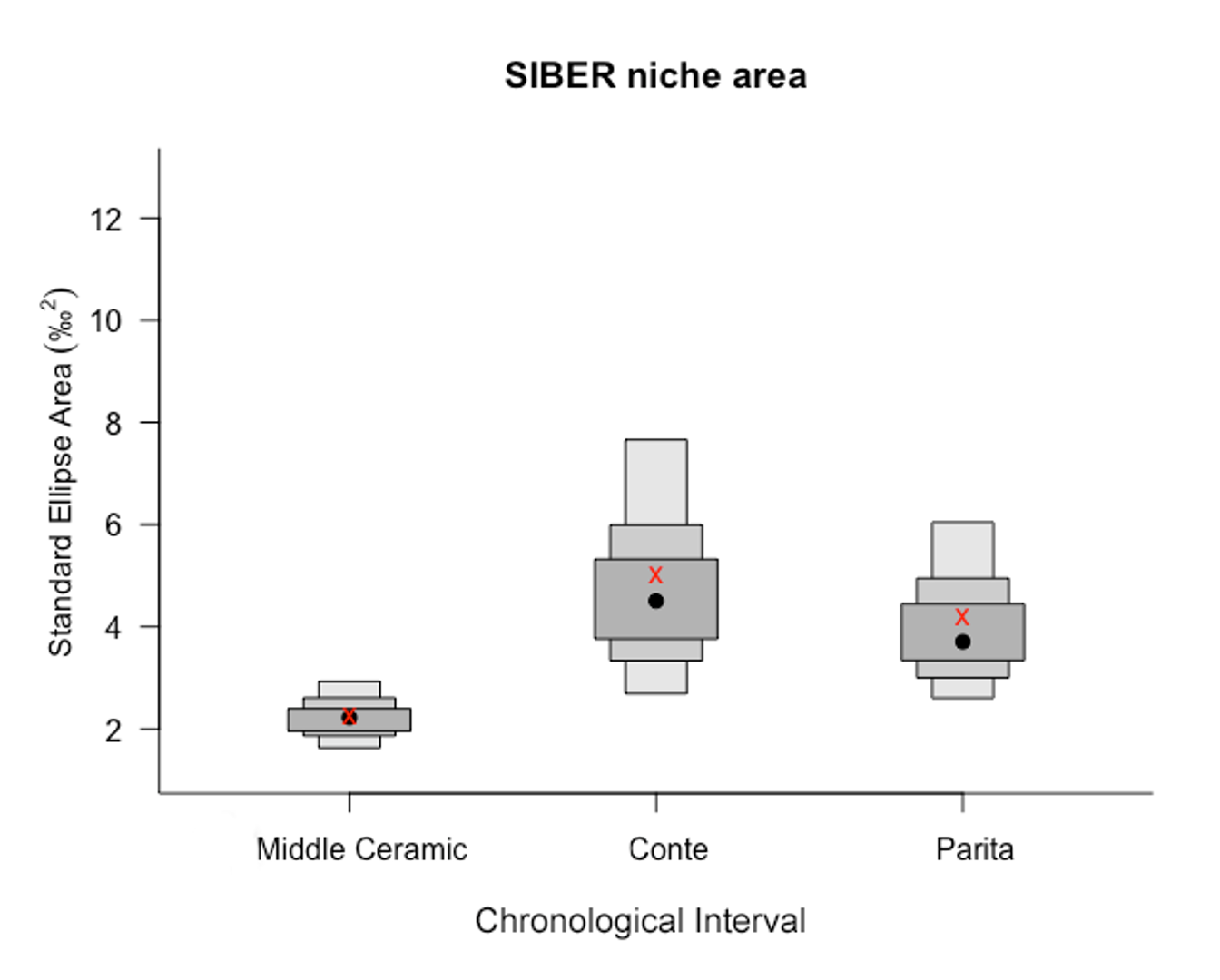

Supplement: S6 Fig — Box plots are the Bayesian results from 20,000 model iterations after 1,000 were thinned. Points represent Bayesian modes, red crosses are means, and the boxes represent the 50%, 75%, and 95% credible intervals, respectively. (TIF) [file pone.0335678.s006.tif]
